# Teaching about the birth of synchrotron light: the role of Frascati and a missed opportunity

**DOI:** 10.1107/S160057752400300X

**Published:** 2024-05-21

**Authors:** G. Margaritondo

**Affiliations:** a Faculté des Sciences de Base, Ecole Polytechnique Fédérale de Lausanne, Switzerland; b Istituto Italiano di Tecnologia Via Morego 30 16163GenovaGE Italy; Bhabha Atomic Research Centre, India

**Keywords:** synchrotron radiation history, Frascati, first synchrotron light projects, X-rays

## Abstract

The users of synchrotron light are now tens of thousands throughout the world. Paradoxically, many of them do not know much about the early history of their domain. This is regrettable, since education about the initial developments makes it easier to fully understand synchrotron radiation and effectively use its amazing features. Scarcely known, in particular, is the key role of scientists working in Frascati, Italy. Partly based on his personal experiences, the author reports here relevant aspects of this story, including a pioneering French–Italian experiment that started in the early 1960s, and the Frascati contributions in the 1970s and 1980s to the birth of synchrotron light research. Finally, the unwise strategic decisions that prevented Italy from achieving absolute leadership in this domain – in spite of its unique initial advantages – are analyzed.

## Abstract 2

A collection of personal reminiscences illuminates many widely unknown facts about the first steps of synchrotron radiation research; in particular, the events that brought Frascati close to world leadership in this domain.

## 1. Foreword

On 8 August 2023, there was a grave loss for the synchrotron light community: its pioneer Emilio Burattini (Burattini, 2016 ▸; Cimino *et al.*, 1992 ▸; Burattini & Simonetti, 1992 ▸; Burattini *et al.*, 1992 ▸, 1995 ▸). I was personally touched, since Emilio and I had worked together in Frascati during the early history of our domain. With him, we also lost important information on that period: only a fraction is preserved in publications and other records (Burattini, 2016 ▸; Cimino *et al.*, 1992 ▸). This made me think about the responsibility to safeguard the historical memories of our field and pass them to the next generations of users.

I thus decided to report several of my own reminiscences – including scarcely known but important facts, some also involving Emilio. This is of course only a partial account of our history, but ‘*meglio poco che niente*’!

### 1.1. Introduction

I recently treated several facets of the initial development of synchrotron light (Margaritondo, 2022 ▸), correcting widespread misconceptions. However, I did not sufficiently describe one important fact: physicists operating in Frascati, Italy, were key protagonists of this history.

Their role materialized in two ways and during two different time periods. In the 1960s, Frascati hosted a pioneering French–Italian synchrotron light experimental project – certainly the first in Europe and one of the earliest in the world (Bonnelle & Dhez, 2014 ▸; Cauchois *et al.*, 1963*a* ▸,*b* ▸; Jaeglé *et al.*, 1967 ▸, 1968 ▸, 1971 ▸). Second, starting in the early 1970s a permanent synchrotron light program became active (Balzarotti *et al.*, 1970 ▸, 1974*a* ▸,*b* ▸,*c* ▸; Tullio & Balerna, 2008 ▸; Preger & Murtas, 1997 ▸; Savoia, 1988 ▸), temporarily putting Frascati ahead of its competitors abroad.

Many outstanding Italian and non-Italian scientists were involved in these enterprises. Regrettably, I cannot mention here all of them. But I welcome the opportunity to present at least some, since their merits are not sufficiently appreciated today.

The role of Frascati in the birth of synchrotron radiation was facilitated by a uniquely fertile environment, created by multiple factors. First, the presence of top talents in electron accelerators. Second, very advanced facilities: Frascati actually pioneered storage rings, the most effective synchrotron light sources. And it could also count on a strong counterpart, the blossoming condensed-matter community in Rome with its world-level physicists.

Within this positive background, a specific development in the mid-1970s created an excellent opportunity for Italy to actually become the world leader of synchrotron light. This development concerned the Frascati storage ring Adone – a superb machine and, potentially, the best light source in the world. Its importance for elementary particle research sharply declined after 1974. Thus, it could have been rapidly converted into a full-time, dedicated synchrotron radiation facility, beating all competitors.

However, we shall see that the conversion was delayed for years. And then it was only partial, dissipating the initial advantages.

## 2. Strong foundations

Let us take a step back to learn how Frascati had become, in the 1950s and 1960s, a front-runner in particle accelerators. The roots of this process had been established two decades before, with the leading neutron research of Enrico Fermi’s team at the University ‘La Sapienza’ (Rome I). Created in 1927, this group involved directly or indirectly exceptional physicists such as Edoardo Amaldi, Emilio Segrè, Ettore Majorana, Enrico Persico, Franco Rasetti and Bruno Pontecorvo. Sadly, it was destroyed in 1938 by the outrageous racial laws of fascism, which forced Fermi and several others to flee from Italy.

Abroad, Fermi was a leader in two strategic enterprises: the first nuclear reactor and the Manhattan Project. The atomic bombs that ended World War II made him (like Oppenheimer and Segrè) also widely known to the general public.

**Figure 1:**
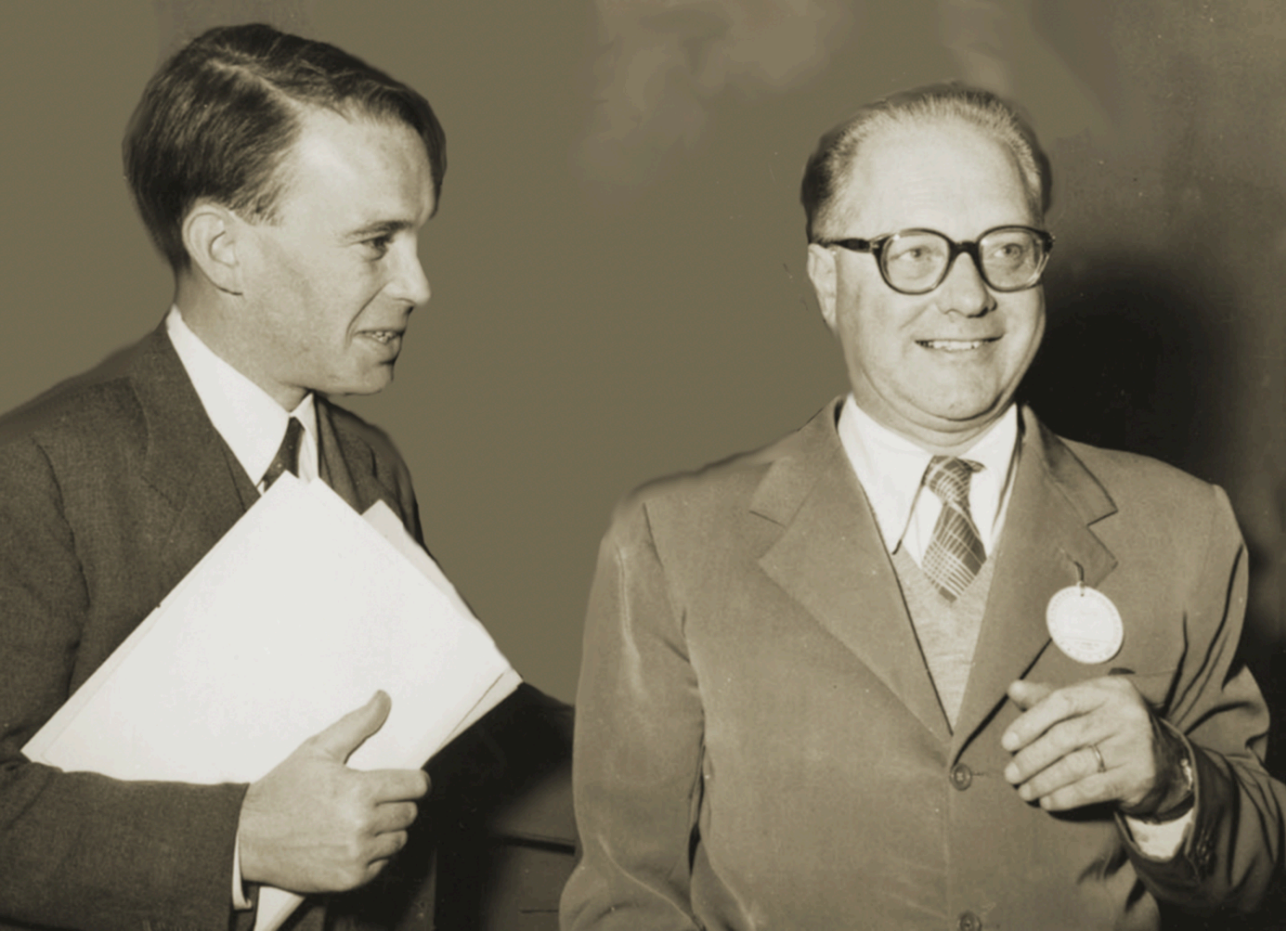
Edoardo Amaldi (right) and Bruno Toushek in the mid-1950s. The images are artist’s rendition portraits created by the author.

**Figure 2:**
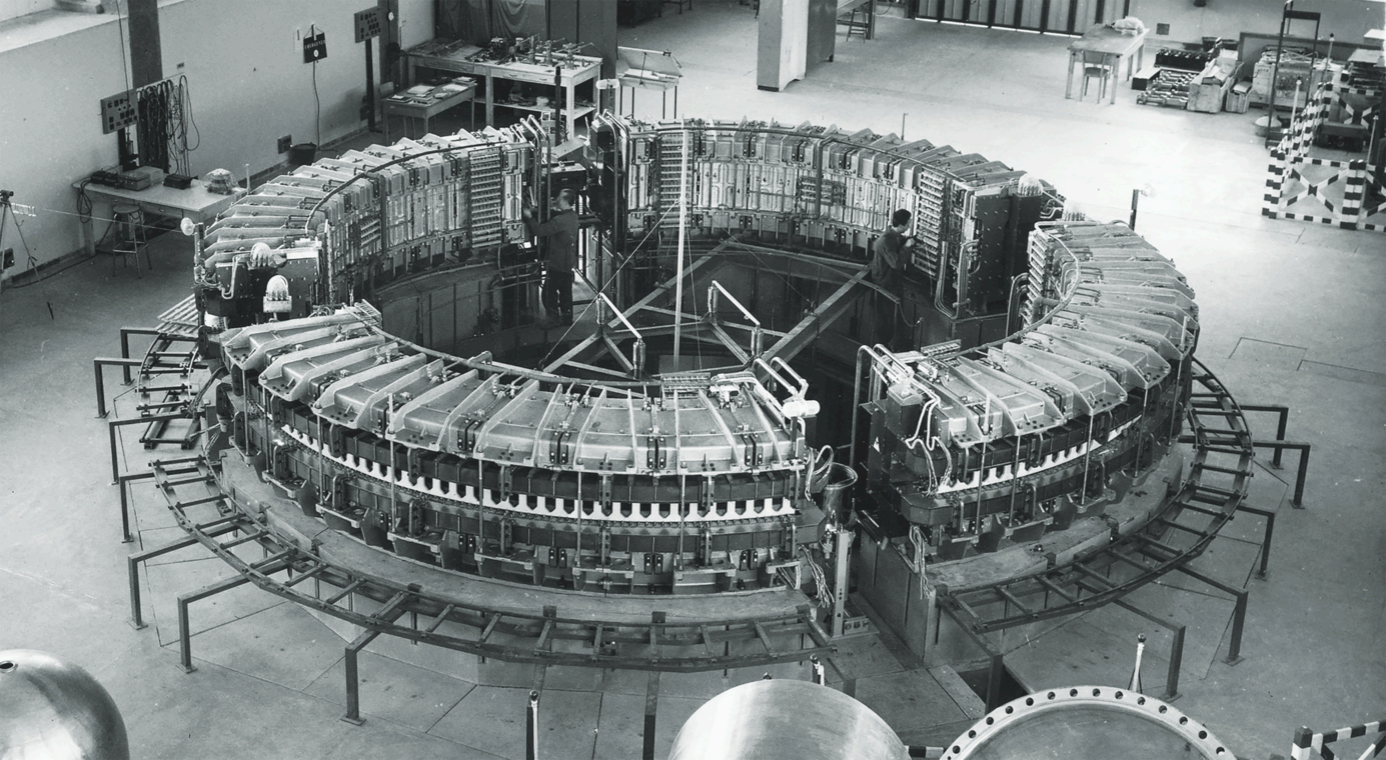
The 1.1 GeV ‘Elettrosincrotrone’ of Frascati, where the Italy-based contributions to synchrotron light history started. Image copyright: INFN.

This fame had a beneficial impact on Edoardo Amaldi’s (Fig. 1 ▸, right) efforts – started in the late 1940s – to reconstruct the Roman physics from the ashes left by racial persecutions. He exploited the strong public image of nuclear research to obtain resources and launch new enterprises, such as the creation in 1951 of INFN (Istituto Nazionale di Fisica Nucleare) and in 1954 of its Laboratori Nazionali in Frascati, where the first advanced particle accelerator in Italy was inaugurated in 1959: a 1.1 GeV electron synchrotron (‘Elettrosincrotrone’) (Fig. 2 ▸).

**Figure 3:**
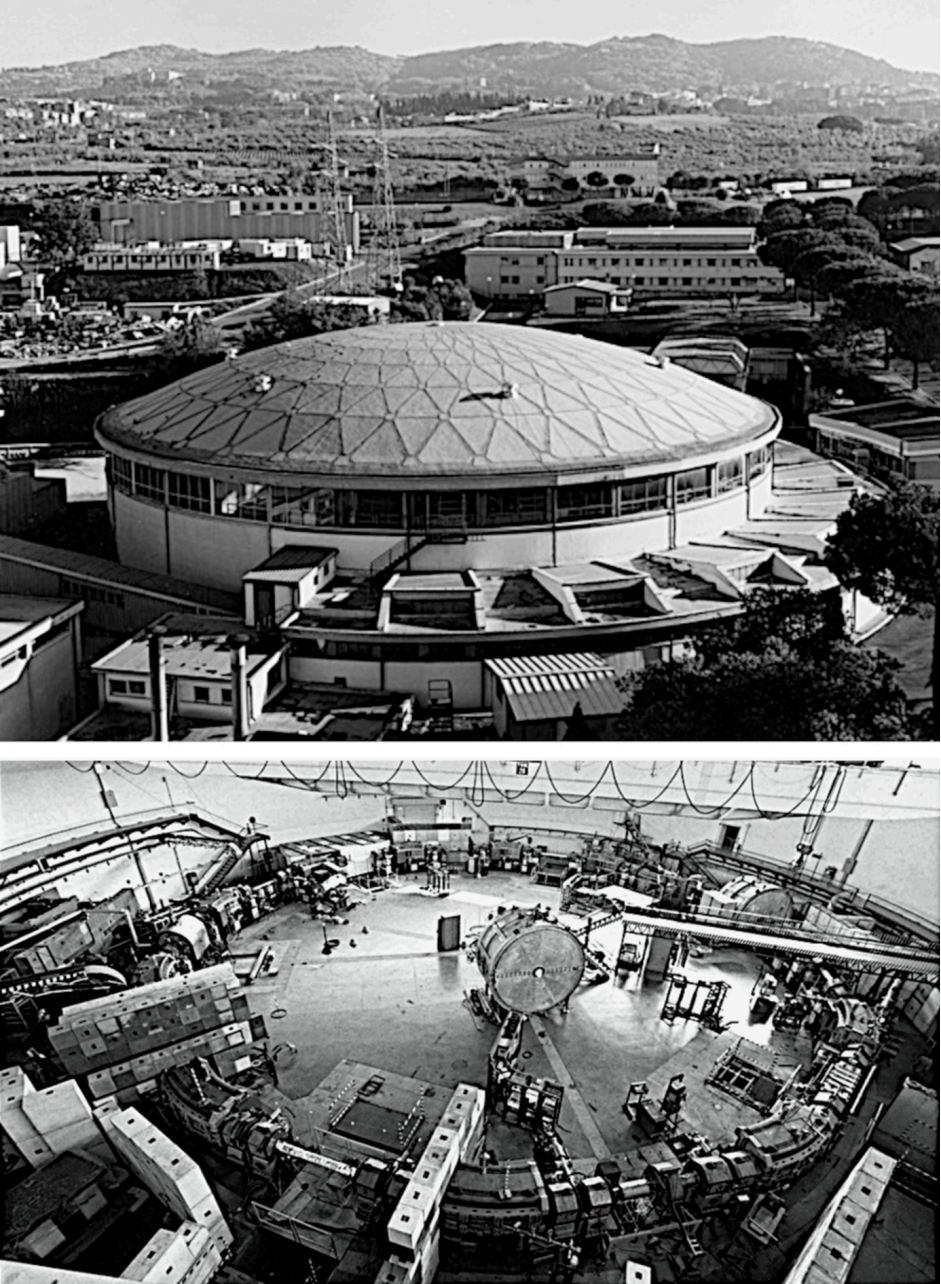
The storage ring Adone – an excellent successor of the ‘Elettrosincrotrone’ – and its beautiful dome. Image copyright: INFN.

Involved in this ambitious project and in other Frascati enterprises were top experts in accelerator science and engineering. Most notably, Amaldi hired in 1953 the Austrian Bruno Toushek (Fig. 1 ▸, left), possibly the best accelerator scientist of all time (Margaritondo, 2021 ▸; Amaldi, 1981 ▸; Bonolis, 2005 ▸; Bonolis & Pancheri, 2011 ▸; Bonolis *et al.*, 2019 ▸; Bernardini, 2004 ▸; Bernardini *et al.*, 2015 ▸). Toushek was a pioneer of the new accelerator trends: storage rings and colliding beams. In 1960, he led (Bernardini *et al.*, 1960 ▸, 1962 ▸) the commissioning in Frascati of the first storage ring in the world, AdA (officially, Anello di Accumulazione – but allegedly named after Toushek’s aunt!). In 1969, AdA was succeeded by its outstanding 3.0 GeV offspring Adone (Fig. 3 ▸).

Objectively, with these achievements Frascati and its accelerators were second-to-none worldwide (Bonolis *et al.*, 2019 ▸). The particle physicists used Adone for front-line experiments, notably with colliding electron–positron beams, and profited from the partnership with outstanding theorists based in Rome.

Unfortunately, these efforts were struck by very bad luck. The energy of Adone was just below the required level for the top result of that period: the J/ψ particle – discovered in 1974 at Stanford and Brookhaven – which validated an essential piece of the developing quark model, the ‘charm quark’ predicted by several theorists including the Italian Luciano Maiani.

**Figure 4:**
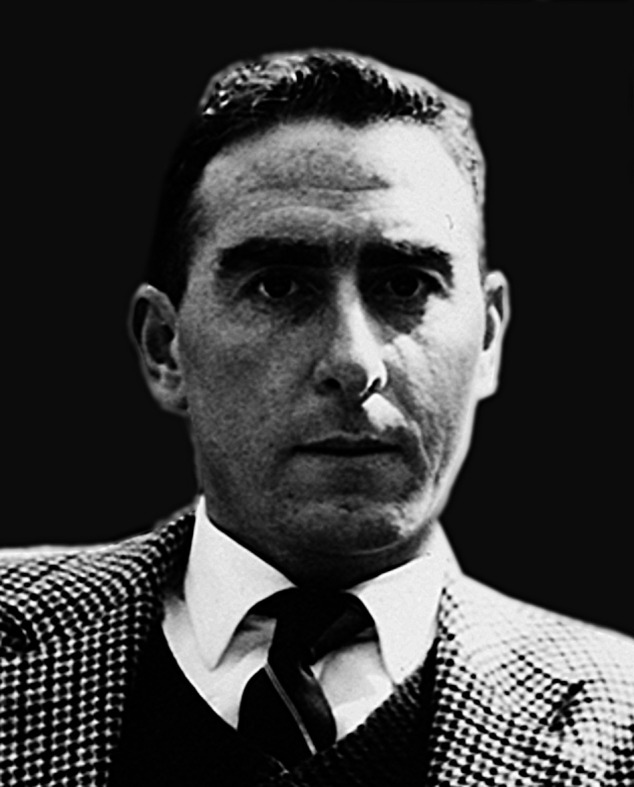
Giorgio Salvini, a key Adone leader, in the mid-1960s. The image is an artist’s rendition portrait created by the author.

**Figure 5:**
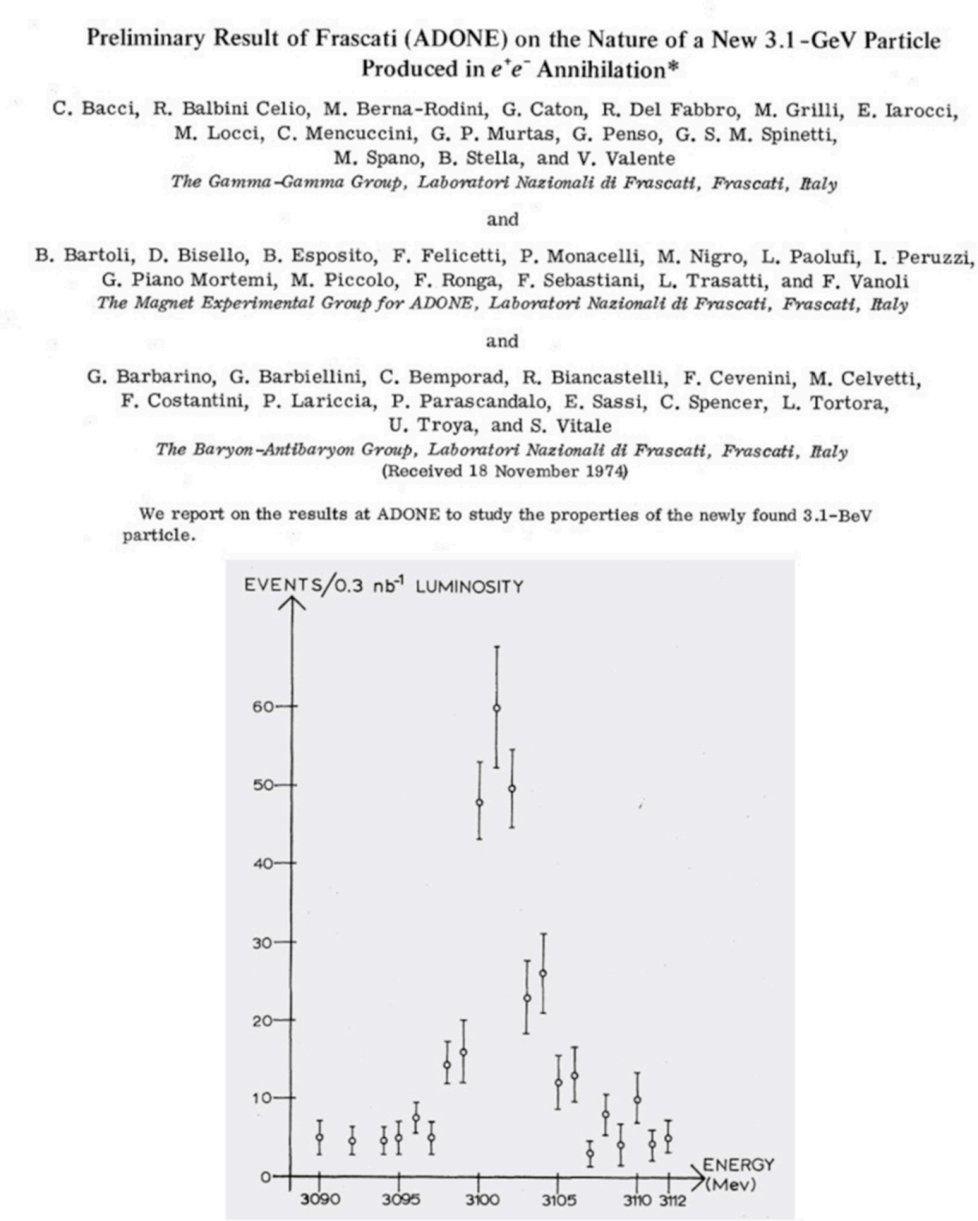
The best performance in the history of Adone: the very fast confirmation in 1974 of the existence and characteristics of the new J/ψ particle (Bacci *et al.*, 1974 ▸). Note the modesty of Salvini and Toushek, who are not even listed as authors.

Frascati did a fantastic job confirming the existence and basic properties of the new particle, after upgrading *in two days* the Adone energy. As I was then working in Frascati, I could witness the justified excitement of my colleagues for this exceptional result. One night in November 1974, walking by the office of the Adone leader and former Frascati director Giorgio Salvini (Fig. 4 ▸), I could hear him dictating to the journal editors, *on the phone*, the experimental points from Adone for a super-fast publication (Bacci *et al.*, 1974 ▸) (Fig. 5 ▸).

The excitement, however, was quickly replaced by depression, as the Frascati scientists realized that they had missed the top prize. Afterwards, the Adone impact on elementary particle research progressively declined. And the efforts to replace it with a more advanced Frascati accelerator, SuperAdone, led to nothing. At any rate, the European center of gravity of particle physics was shifting to CERN in Geneva, Switzerland, launched by Amaldi and other leading physicists, and then developed with very strong Italian contributions. Adone was finally decommissioned in 1993.

## 3. The parallel development of synchrotron light

**Figure 6:**
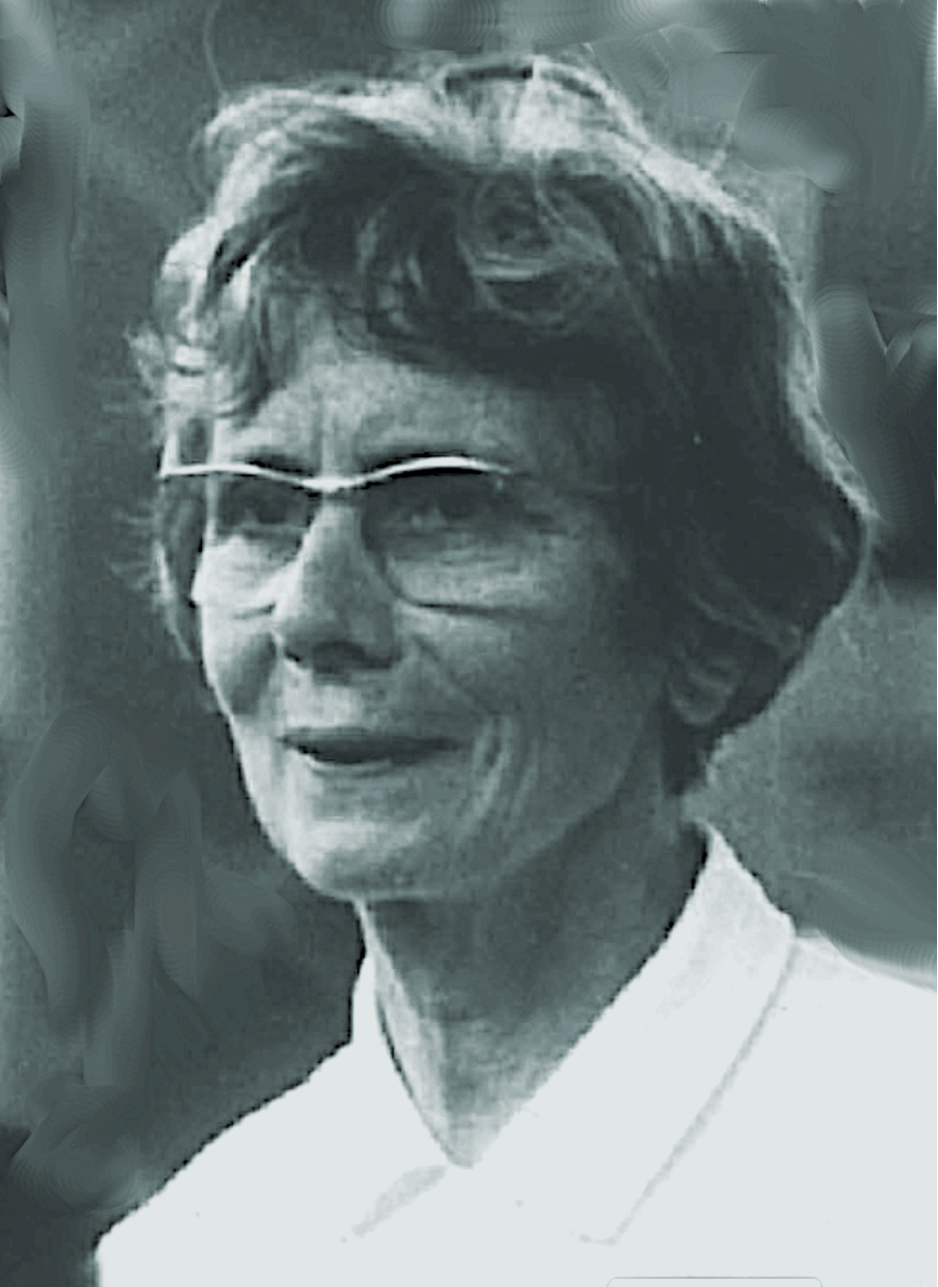
Yvette Cauchois, the scientist who pioneered synchrotron light experiments in Frascati – and in the world. The image is an artist’s rendition portrait created by the author.

Independent of the above events, and scarcely known by most of their protagonists, there was another important development in Frascati: synchrotron light research, initiated in 1961 under the leadership of Yvette Cauchois (Fig. 6 ▸), director of the Laboratoire de Chimie Physique at the Sorbonne.

Cauchois had learned about short-wavelength synchrotron radiation from her friends Parrat (Parratt, 1959 ▸) and Tomboulian (Tomboulian & Hartman, 1956 ▸; Tomboulian & Bedo, 1958 ▸) of Cornell. A leading X-ray spectroscopist, Cauchois wanted to perform experiments with the emission and realized that the Frascati ‘Elettrosincrotrone’ was a suitable source. A Sorbonne–Frascati collaboration was also promoted by the eminent physicist Ugo Fano, resident in the USA but very influential in Italy.

The emission of electromagnetic waves by accelerated charged particles had actually been known for a very long time, starting from Maxwell’s electromagnetism theory (Maxwell, 1865 ▸, 1873 ▸), and it had become a hot topic in the 1930s, due to the advent of electron accelerators like the cyclo­tron and the betatron (Widerøe, 1928 ▸; Lawrence & Edlefsen, 1930 ▸; Kerst, 1941 ▸). Indeed, the emission of synchrotron radiation strongly influenced the operation of these machines.

The issue had become even more important with the invention of synchrotrons by Veksler and McMillan (Veksler, 1944 ▸; McMillan, 1945 ▸), and had stimulated the pioneering theoretical contributions of Soviet Union scientists led by Pomeranchuk and Ivanenko (Iwanenko & Pomeranchuk, 1944 ▸), followed in the USA by Julian Schwinger’s pivotal description (Schwinger, 1949 ▸) of the different aspects of the emission of radiation by accelerated relativistic electrons.

However, the practical use of the radiation – in particular of its ultraviolet and X-ray components – had not been initially considered. In fact, the emission of potentially useful short wavelengths was largely unknown. Classical physics predicted instead long wavelengths not far from the size of the accelerator magnets and corresponding to the ‘cyclo­tron frequency’ of the electron motion. As a consequence, the first efforts to detect synchrotron radiation had targeted radio waves (Margaritondo, 2022 ▸), and the observation in 1947 of shorter-wavelength visible synchrotron light was a lucky accident.

**Figure 7:**
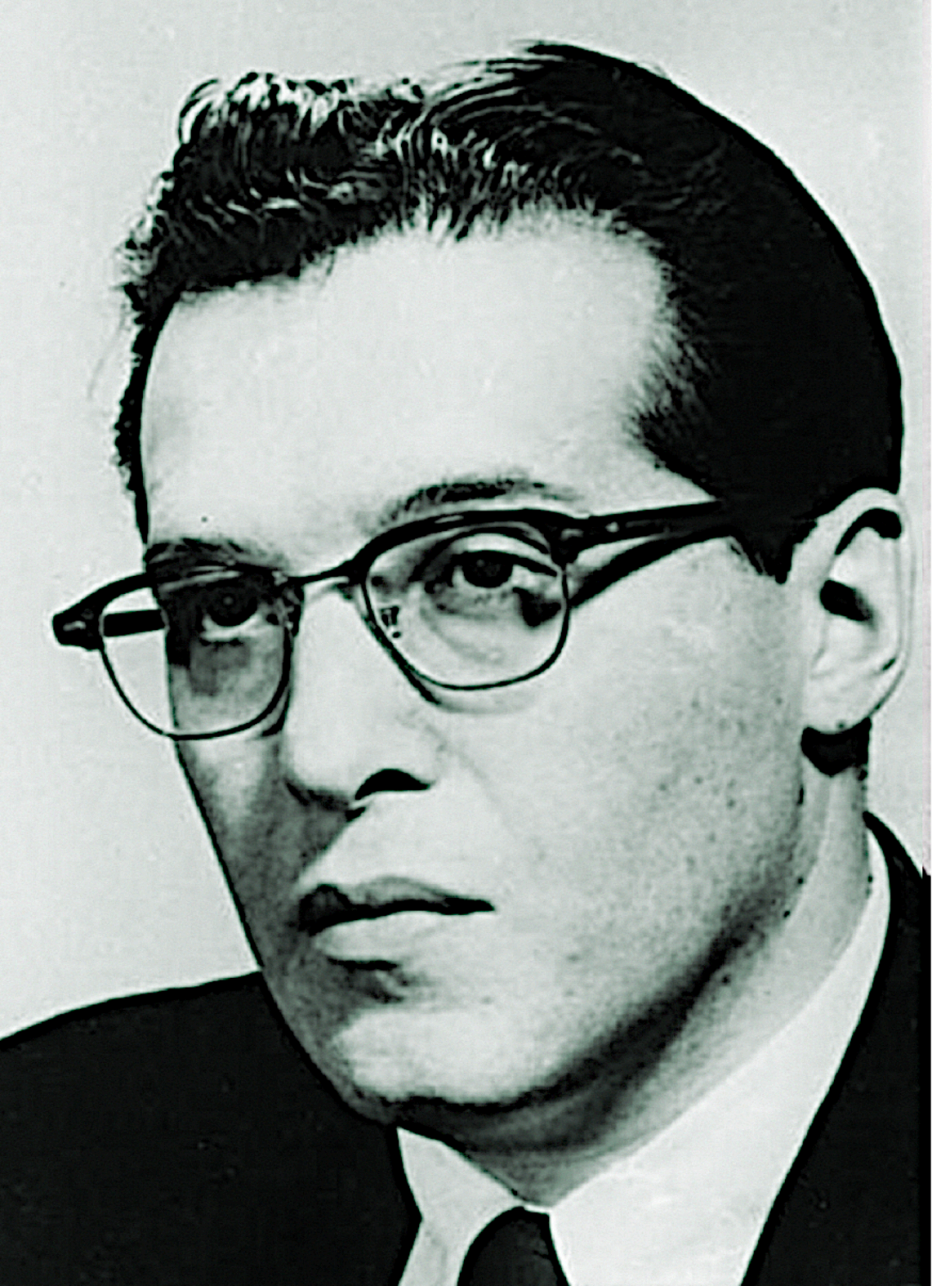
The theorist Julian Schwinger, who predicted the short wavelengths of synchrotron radiation from relativistic electrons. The image is an artist’s rendition portrait created by the author.

As it is known, the spectrum of synchrotron radiation from high-energy electrons is determined by relativity, which notably allows the production of short wavelengths, and it had been theoretically predicted in the 1940s by Julian Schwinger (Fig. 7 ▸). But he was slow in communicating his results, using for years conference presentations before a full article (Schwinger, 1949 ▸). His theory was very complicated, hindering dissemination beyond a handful of accelerator experts.

This is why it took a decade for the news of short wavelengths from relativistic electrons to reach Yvette Cauchois. Only much later it was realized (Margaritondo, 2017 ▸; Hwu & Margaritondo, 2021 ▸) that synchrotron emission can be understood with very simple relativistic arguments and no complicated formalism at all.

**Figure 8:**
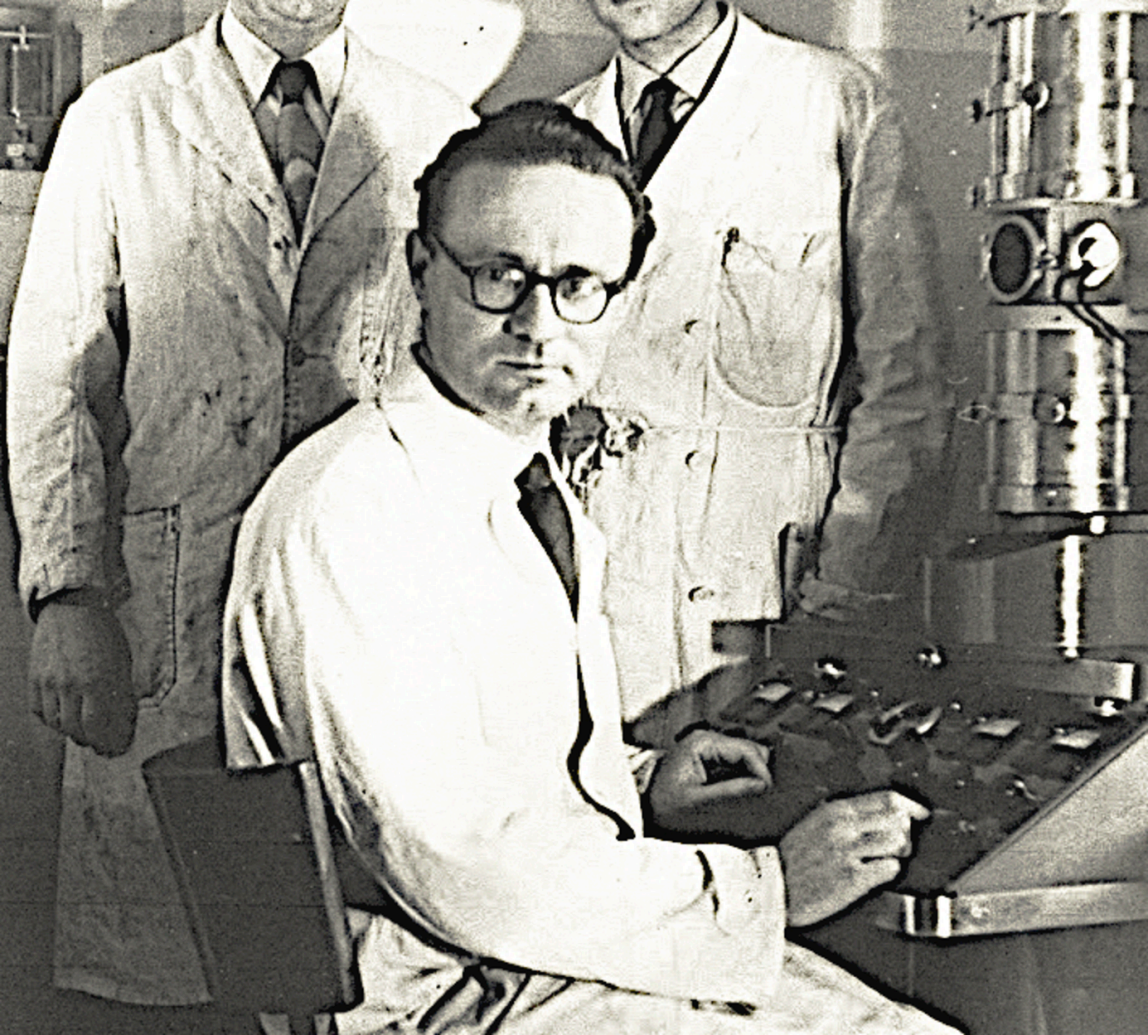
Mario Ageno, illuminated Italian partner of Yvette Cauchois. The image is an artist’s rendition portrait created by the author.

**Figure 9:**
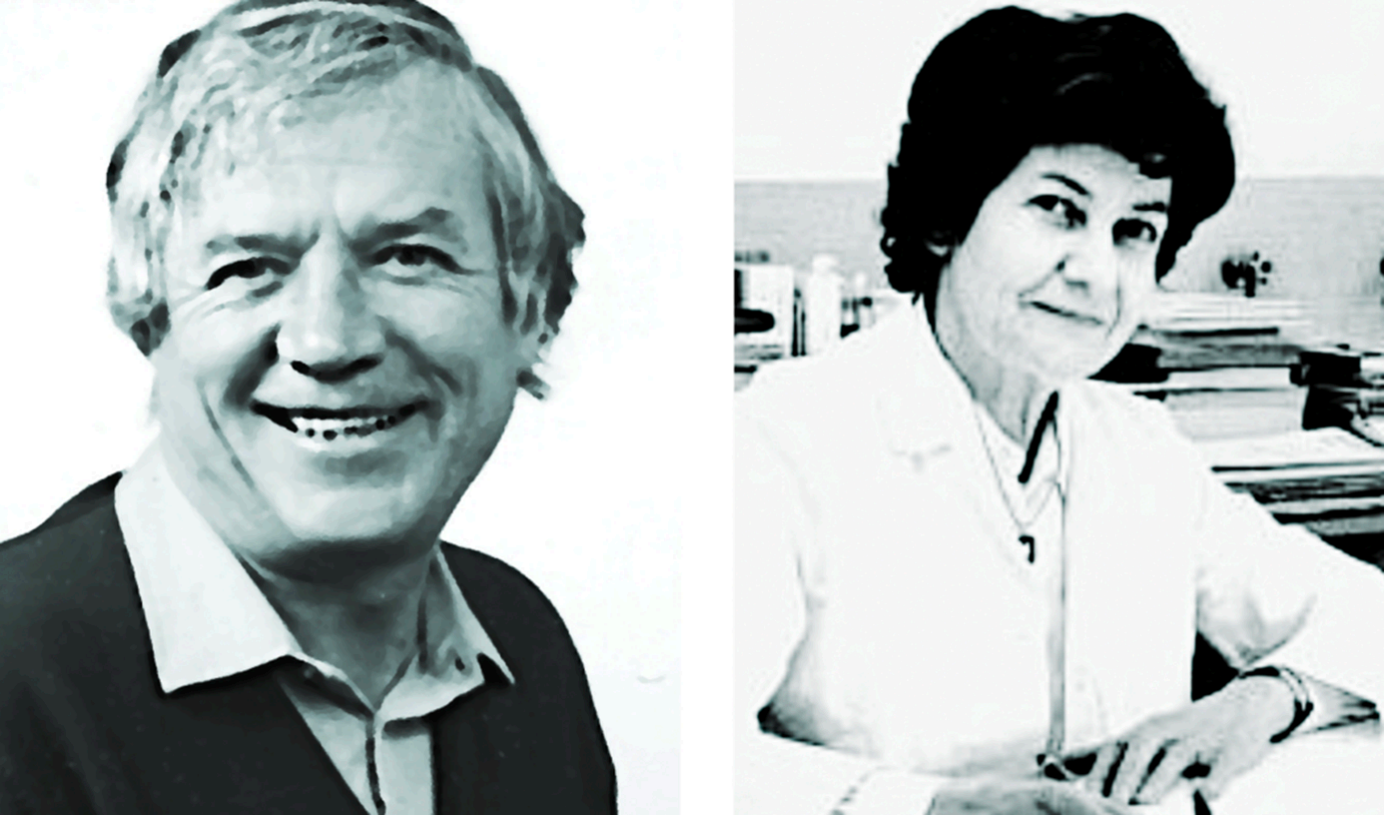
Cauchois’ collaborators Pierre Jaeglé and Christiane Bonnelle. The images are artist’s rendition portraits created by the author.

On 17 March 1961, Cauchois wrote to Mario Ageno (Fig. 8 ▸) of the Istituto Superiore di Sanità (ISS) in Rome, proposing joint experiments. Ageno, being an excellent physicist, immediately resonated to the idea: Cauchois received his positive response on 28 March. She visited Frascati in September with her associates Pierre Jaeglé and Christiane Bonnelle (Fig. 9 ▸), and the collaboration started shortly afterwards (Bonnelle & Dhez, 2014 ▸; Cauchois *et al.*, 1963*a* ▸,*b* ▸; Jaeglé *et al.*, 1967 ▸, 1968 ▸, 1971 ▸). Pierre Dhez, Françoise Combet-Farnoux and Yvonne Héno joined the French contingent. On the Italian side, the cooperation involved Guido Missoni and later Marta Cremonese and Giuseppe Onori, all of the ISS.

**Figure 10:**
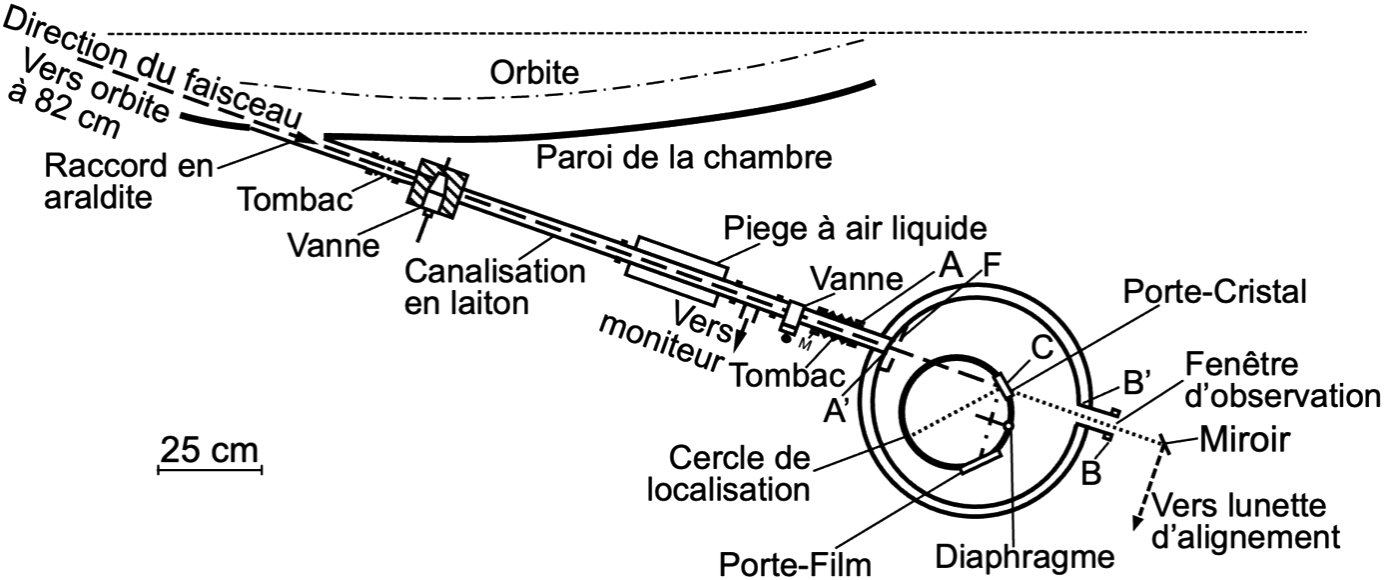
Scheme drawn by the author of the apparatus used for some of the first experiments of ‘Sanità Luce’ (Cauchois *et al.*, 1963*a* ▸,*b* ▸).

The project, called ‘Sanità Luce’, obtained the first data (Cauchois *et al.*, 1963*a* ▸,*b* ▸) in the spring of 1963, using two spectrometers (one named after Cauchois herself). The detection was performed with photographic plates read with a densitometer. X-ray absorption was measured around the *K*-edges of different metal specimens (Figs. 10 ▸ and 11 ▸).

This was objectively an epochal event, but, incredibly, it went almost unnoticed. Indeed, it was not presented in high-visibility journals but first communicated in Italian at the 1963 annual conference of the Società Italiana di Fisica, and then reported in French in the *Comptes Rendus* (Cauchois *et al.*, 1963*a* ▸,*b* ▸).

Why this low-key dissemination? Because these first experiments were not really solid-state studies but investigations of the properties of synchrotron light itself. Indeed, Schwinger’s theory (Schwinger, 1949 ▸) had not yet been fully digested, and ‘Sanità Luce’ preferred to experimentally verify its predictions before embarking in a complex project.

Its first results achieved this goal, specifically showing that the radiation beam divergence changed with the electron energy as theoretically predicted (Schwinger, 1949 ▸). The experiments did detect important solid-state features such as X-ray absorption edges. Thus, they were a milestone in the birth of synchrotron radiation – which would have deserved a more effective dissemination!

The ‘Sanità Luce’ experiments continued for years, being extended to different spectral regions and also to gas-phase phenomena. They were not, therefore, a one-shot affair but a long-term program – which lasted until 1971 when Cauchois moved her activities to the storage ring ACO in Orsay. Its protagonists, therefore, merit a long-lasting fame.

Cauchois herself is well known in France but not sufficiently elsewhere. And her Italian partners are scarcely remembered even in their own country – whereas they should be known and celebrated. Let us learn a bit more, at least about the two most prominent ones.

Mario Ageno (Fig. 8 ▸) had graduated as one of Fermi’s last students in Rome. He was then involved in accelerators as a collaborator of Edoardo Amaldi. Afterwards, he became the first Italian biophysicist – author of important results, notably about bacterial growth. In 1959, he was nominated director of the ISS Physics Department and, in 1969, appointed at the University of Rome I to the first Italian biophysics chair.

Unfortunately, his research did not find an effective interface in the local biomedical community and remained mostly isolated. But Ageno was posthumously recognized, at least in part: a Roman street is now named after him.

Guido Missoni was a remarkable experimentalist. His own narration (Missoni, 2017 ▸) shows that he was a pillar of ‘Sanità Luce’. And his other diversified research contributions were also quite important.

His institution, the ISS, was charged to monitor radiation in different applications: Missoni developed reference standards and the corresponding instrumentation. This work had an important impact on the medical use of X-rays. Quoting again from him (Missoni, 2017 ▸): ‘*It was not unusual to find cases of radiology analysis in which the procedure and the doses used to obtain the images were delivering huge and unnecessary radiation quantities to the patients and, above all, to the operators*’. Missoni’s work contributed to the elimination of such malpractices.

He also obtained important results on radioactive tracers. The most significant were water tracing at the site of the Vajont dam disaster and at the Timavo river near Trieste. Therefore, Missoni combined his key contributions to the birth of synchrotron light with an eminent applied-physics career, whose societal impact was remarkable.

## 4. The next phase

Not long after the end of ‘Sanità Luce’, a new long-term synchrotron light program became active in Frascati. Initially called ‘Solidi Roma’ (Balzarotti *et al.*, 1970 ▸, 1974*a* ▸,*b* ▸,*c* ▸), its name was later changed to ‘PULS’ (Programma Utilizzazione Luce di Sincrotrone) (Tullio & Balerna, 2008 ▸; Preger & Murtas, 1997 ▸; Savoia, 1988 ▸). To understand its background, we must consider the evolution of solid-state physics in Rome.

Before the 1950s, there had not been much condensed-matter research in the Roman institutions (nor elsewhere). Indeed, solid-state physics had been recently born as an offspring of quantum mechanics. One should not forget, however, that a major initial result had been the Fermi–Dirac statistics, formulated shortly before Fermi’s faculty appointment in Rome.

**Figure 12:**
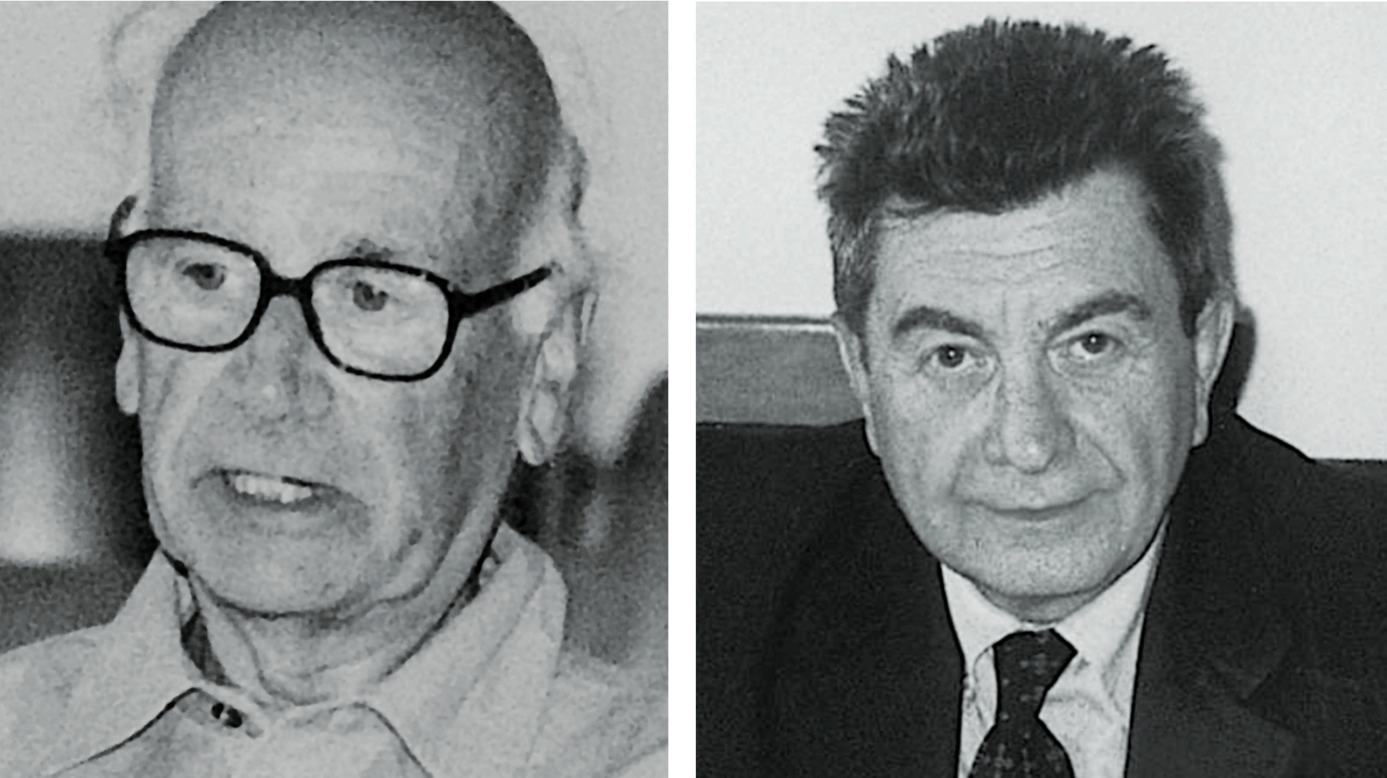
Gianfranco Chiarotti (left) and Franco Bassani, leaders of the permanent Frascati synchrotron light program that was initially called ‘Solidi Roma’ and later PULS. The images are artist’s rendition portraits created by the author.

Solid-state physics literally exploded in the 1950s, in Italy as in other countries, notably due to its potential applications. Edoardo Amaldi was well aware of this evolution and recruited for the reconstruction of physics in Rome several outstanding condensed-matter scientists, notably including the experimentalists Giorgio Careri and Gianfranco Chiarotti (my future thesis director) and the theorist Franco Bassani (Fig. 12 ▸). With them and their teams, condensed matter in Rome quickly reached a top international stature.

Chiarotti quickly discovered the research opportunities offered by the Frascati accelerators and their synchrotron radiation. However, his projects were affected by events that also strongly damaged Amaldi’s reconstruction of the Roman physics, which started with the university revolt of 1968 and eventually degenerated into terrorism.

The atmosphere of the Rome Physics Department in the 1970s became poisoned by internal political fights that occasionally led to violence. Fed up with the conflicts and the disruption of their activities, several of the top talents that had been attracted by Amaldi decided to leave Rome.

**Figure 13:**
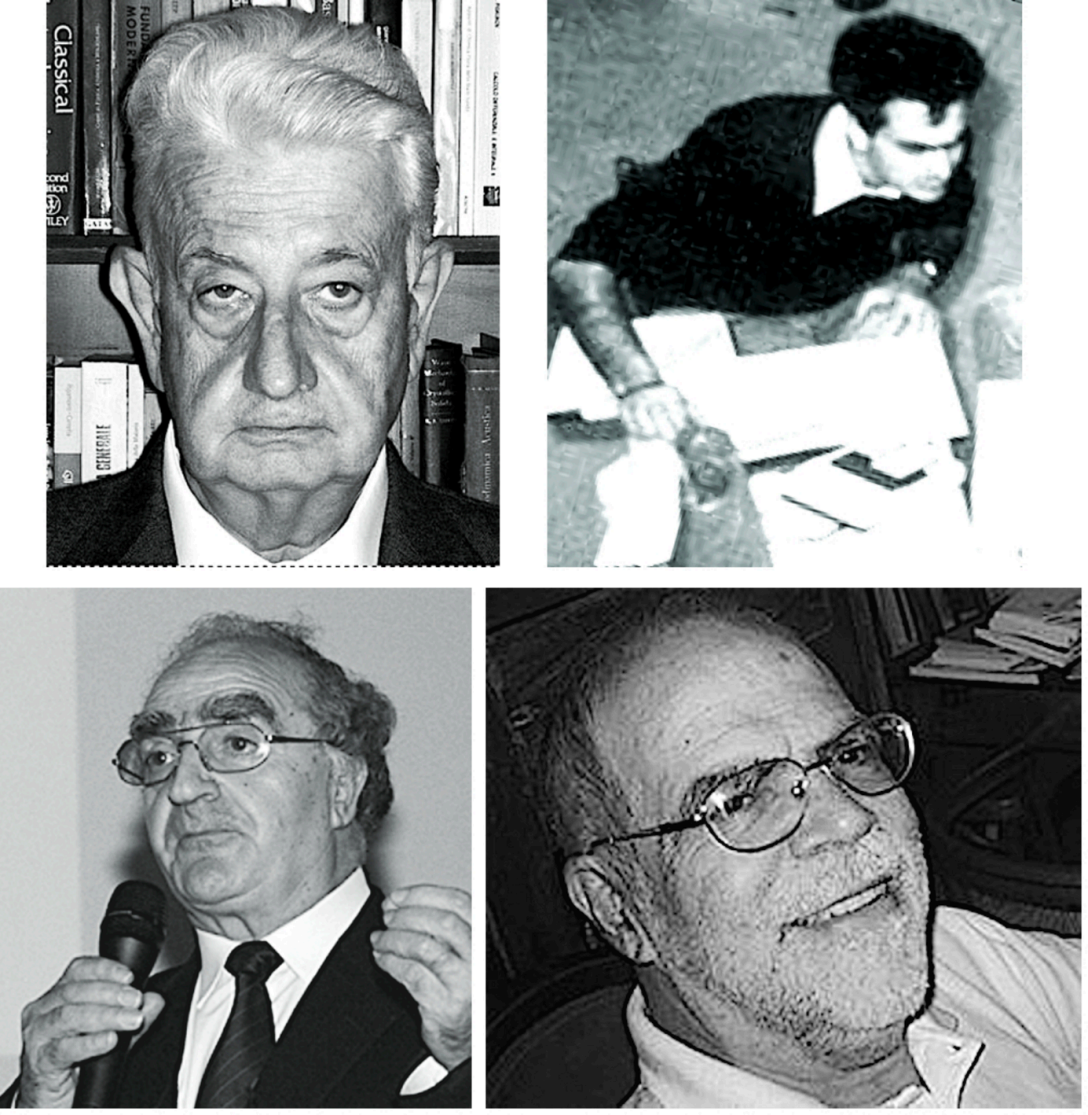
Some early members of ‘Solidi Roma’. Left to right – top: Adalberto (Camillo) Balzarotti and Mario Piacentini; bottom: Emilio Burattini and Antonio Bianconi. The images are artist’s rendition portraits created by the author.

The turmoil also impacted the activities in Frascati. Although facing a very difficult situation, in the early 1970s Gianfranco Chiarotti courageously launched ‘Solidi Roma’ (Balzarotti *et al.*, 1970 ▸, 1974*a* ▸,*b* ▸,*c* ▸), involving members of his ‘La Sapienza’ Physics Department team (Fig. 13 ▸): Adalberto (Camillo) Balzarotti, Mario Piacentini, Emilio Burattini and Antonio Bianconi; plus two collaborators from other institutions: Mario Grandolfo (ISS) and Roberto Habel (INFN). In 1975, they were joined by Andrzej Kisiel, a visiting Polish professor from the Krakow Jagellonian University (Kisiel, 2008 ▸).

**Figure 14:**
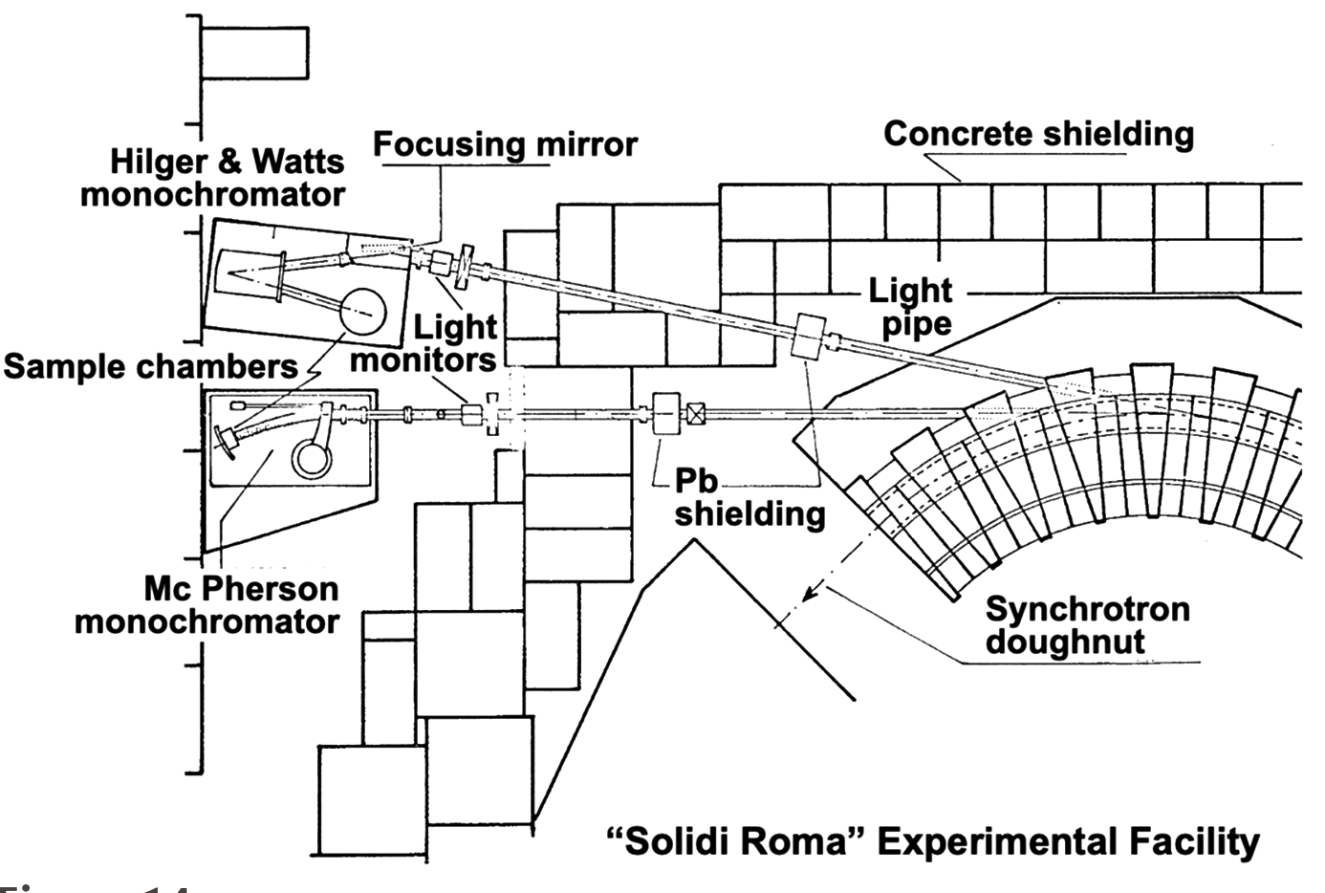
Scheme drawn by the author of a ‘Solidi Roma’ apparatus (Balzarotti *et al.*, 1970 ▸).

**Figure 15:**
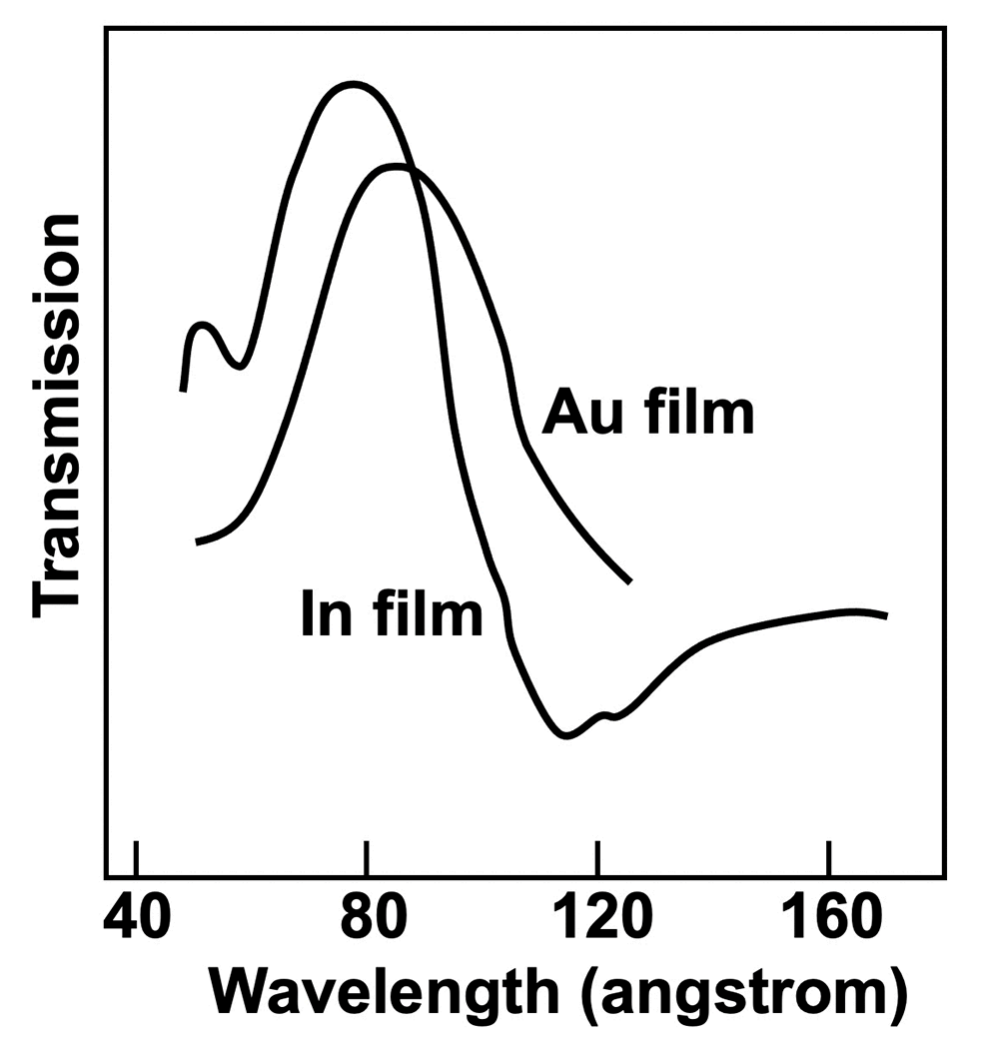
One of the first results of ‘Solidi Roma’. The plot was drawn based on data from Balzarotti *et al.* (1972 ▸).

The primary interest of ‘Solidi Roma’ was ultraviolet and X-ray spectroscopy (Fig. 14 ▸). The team produced a number of excellent results (Balzarotti *et al.*, 1970 ▸, 1974*a* ▸,*b* ▸,*c* ▸), at the world forefront of the still very rare synchrotron light activities (Fig. 15 ▸).

Like those of ‘Sanità Luce’, they were obtained overcoming not only the political turmoil but also tremendous technical difficulties, which were present not only in Frascati but were severely affecting the birth of synchrotron radiation everywhere in the world.

## 5. From synchrotrons to storage rings

What caused such technical difficulties? Primarily, the mode of operation of synchrotrons – which were pulsed machines where electrons were continuously injected and quickly dumped. The control electronics generated tremendous pulses interfering with the devices used for experiments.

Furthermore, the pulsed operation produced very dangerous radiation. Thus, the users could not continuously work close to a synchrotron. Adjustments of the experimental system could only be made during the short idle times between two (long) operation periods of the accelerator.

This slowed down even the simplest tasks, like sample alignment. The duration of a synchrotron light experiment, in Frascati and everywhere else, was orders of magnitude longer than today, jeopardizing productivity and the users’ careers.

I personally witnessed an accident that vividly illustrates the difficulties of using a synchrotron. Frascati had decided to replace the original ‘Elettrosincrotrone’ vacuum chamber with a beautiful new one fabricated using ceramics. After its installation, the pulsed operation restarted. But someone had forgotten to put back in place some of the spacers between magnets, so two poles crashed into each other crushing the new vacuum chamber, which imploded, sending fragments and powder all around the ring – including the experimental beamline that we had just completed. Months of our work were instantaneously nullified.

The technical problems were not only slowing down but *de facto* impeding the birth of synchrotron light research: the field risked an abortion. Fortunately, the difficulties dis­appeared with the advent of storage rings, whose operation is continuous and not pulsed: the electrons circulate in the vacuum chamber for days – or even indefinitely with top-up injections. Thus, storage rings salvaged the entire domain.

**Figure 16:**
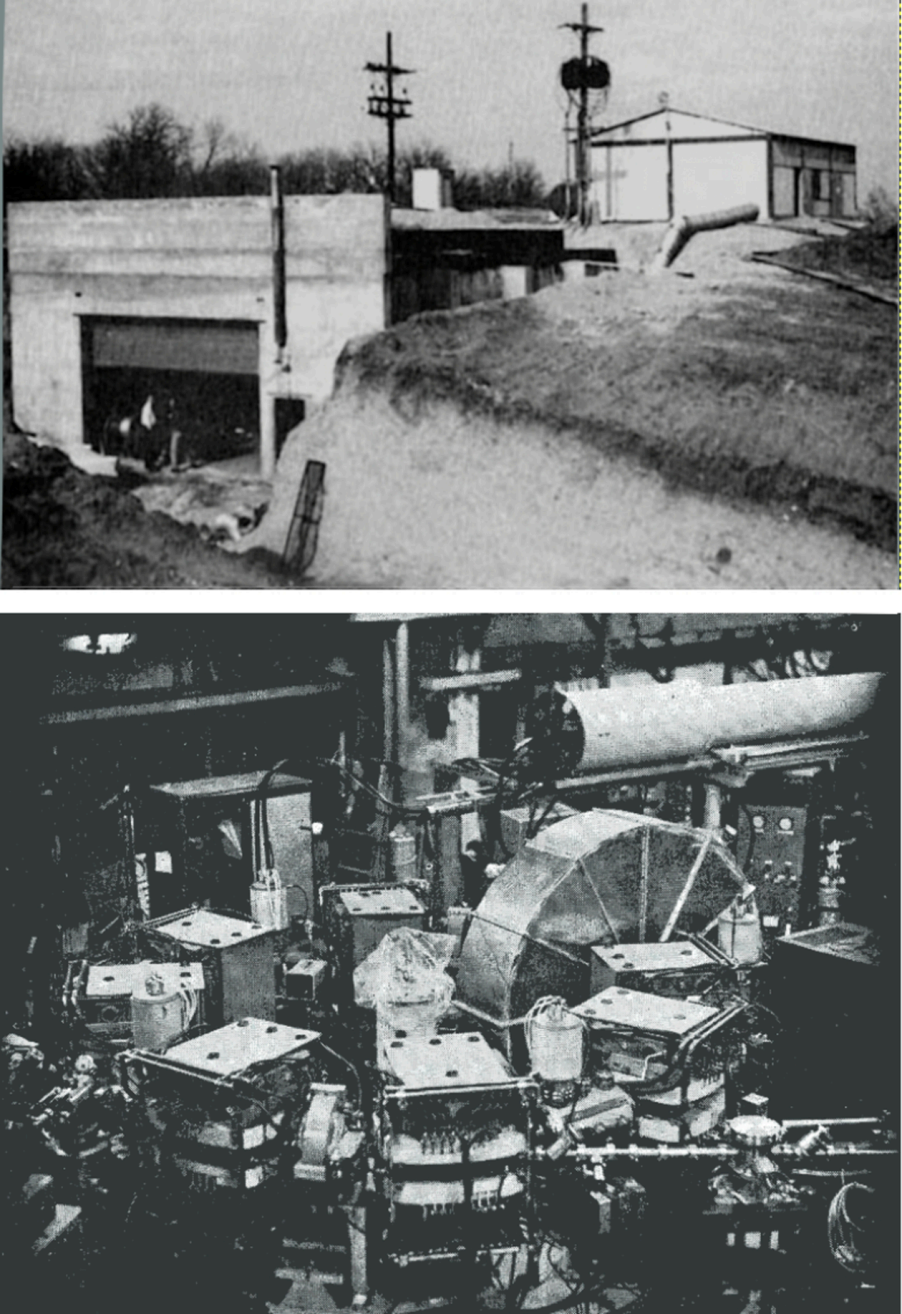
Tantalus in Wisconsin, the first dedicated synchrotron light source in the world. Much less sophisticated than Adone and housed in a shabby cavern rather than in a beautiful building (Fig. 3 ▸), but a great success in the history of synchrotron radiation. The figures are artist’s rendition images created by the author.

Their superiority as light sources was fully demonstrated, starting in 1968, by the practical experience of the 240 MeV Tantalus ring of the University of Wisconsin-Madison (Fig. 16 ▸), which had a peculiar history (Margaritondo, 2008 ▸; Lynch *et al.*, 2015 ▸): it had been built as a demonstration project to promote a coalition of Midwest universities as a legitimate candidate in the competition for the future Fermilab.

**Figure 17:**
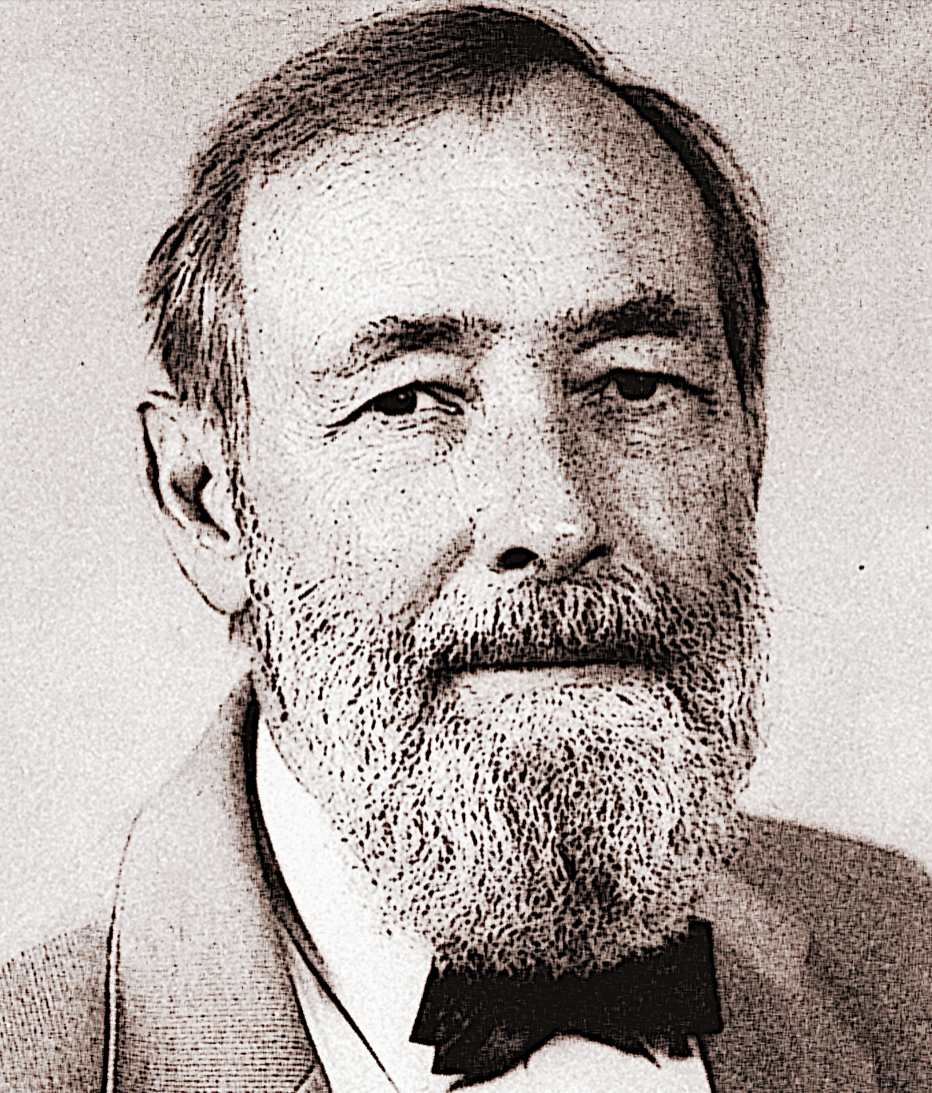
Ed Rowe, father and visionary manager of Tantalus. The image is an artist’s rendition portrait created by the author.

After the coalition lost the race to Illinois, Tantalus no longer had a mission. But an illuminated group of scientists led by its builder Ednor (Ed) M. Rowe (Fig. 17 ▸) made a bold proposal: using it as a fully dedicated synchrotron light source.

The proposal was greeted with almost universal scepticism and faced huge technical and financial problems. In particular, the funding agencies could not believe that synchrotron radiation could attract a half dozen user groups, the minimum needed to justify the investment! However, in spite of the difficulties Tantalus started its operation as a synchrotron source in 1968.

It was a tremendous success, for two reasons. First, it was of course immune from the technical problems affecting synchrotrons. Furthermore, it was fully dedicated to synchrotron light activities. For the first time in history, these were not subject to compromises imposed by the coexistence with other applications – primarily particle research – which negatively affected other facilities.

## 6. The PULS project

**Figure 18:**
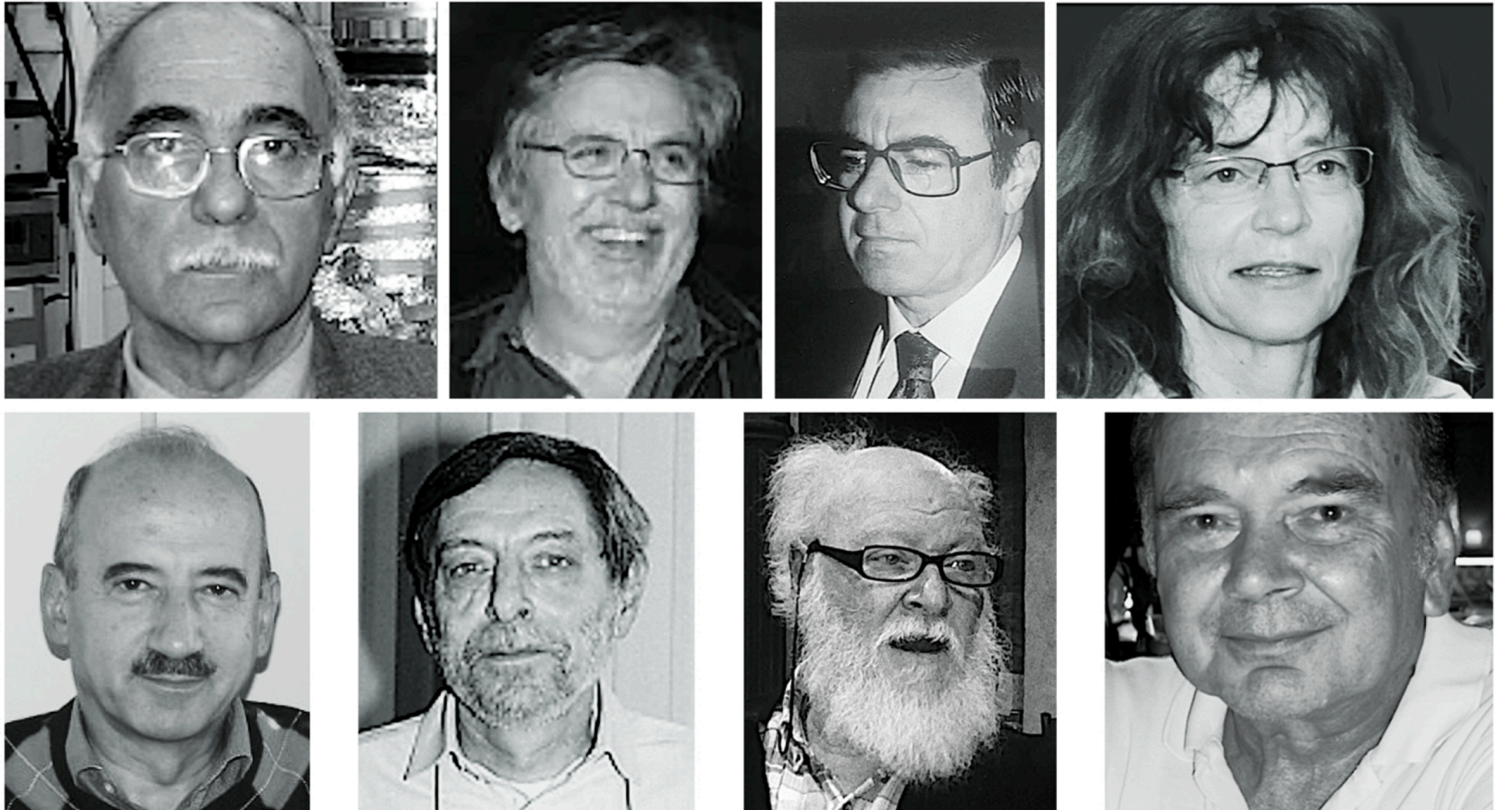
A few of the many scientists and technicians associated with PULS. Left to right – top: Paolo Perfetti, Fabio Comin, Adolfo Savoia, Antonella Balerna; bottom: Settimio Mobilio, Gilberto Vlaic, Mario Capozi, Renzo Rosei. The images are artist’s rendition portraits created by the author.

The success of Tantalus induced Frascati to consider transferring its synchrotron light programs to the superb storage ring Adone. To promote the change, ‘Solidi Roma’ evolved in 1976–77, under the new name PULS (Figs. 18 ▸ and 19 ▸), becoming a collaboration between INFN and the Consiglio Nazionale delle Ricerche (CNR) (Tullio & Balerna, 2008 ▸; Preger & Murtas, 1997 ▸; Savoia, 1988 ▸). Charged with many other responsibilities, Chiarotti transferred the leadership to Franco Bassani.

This also brought a marked change in style. Bassani had no detailed familiarity with experiments, since he was a solid-state theorist – a leading one, in fact, and the founder of a top school. But he had a grandiose vision for PULS and was not timid about soliciting and using the large amounts of money needed for synchrotron radiation activities. Whereas Chiarotti had been somewhat reluctant about requesting and spending large sums.

PULS attracted many young scientists that expanded the initial ‘Solidi Roma’ team – formally joining it, externally collaborating to its tasks or becoming otherwise associated. Among them we can mention Ivano Abbati, Francesco Antonangeli, Antonella Balerna, Maurizio Benfatto, Federico Boscherini, Lucio Braicovich, Cristiano Capasso, Mariangela Cestelli Guidi, Piero Chiaradia, Roberto Cimino, Elio Colavita, Carlo Coluzza, Fabio Comin, Ivan Davoli, Maurizio De Crescenzi, Florestano Evangelisti, Giuseppe Faraci, Adriano Filipponi, Fabia Gozzo, Enrico Gratton, Alfonso Franciosi, Umberto Maria Grassano, Pupa Gilbert, Mario Iannuzzi, Lucia Incoccia, Andrea La Monaca, Augusto Marcelli, Silvio Modesti, Annibale Mottana, Stefano Nannarone, L. Papagno, Fulvia Patella, Agata Pennisi, Maria Novella Piancastelli, P. Picozzi, Claudio Quaresima, Carlo Rizzuto, Renzo Rosei, Nicola Rosato, Felice Rosito, Adolfo Savoia, Augusto Scacco, Stefano Selci, Sergio Stizza, Giancarlo Strinati, Antonio Terrasi, Gilberto Vlaic, Franco Zanini, Nicola Zema. And other scientists, plus excellent technicians such as Mario Capozi, Renato Generosi, Luciano Moretto and Sandro Rinaldi.

The list also included future synchrotron radiation leaders such as Settimio Mobilio and Francesco Sette, the present director general of the European Synchrotron Radiation Facility (ESRF). An entire group was transferred to PULS from LESS (the CNR Laboratorio di Elettronica dello Stato Solido, now Istituto di Elettronica dello Stato Solido) in Rome. This move involved Franco Cerrina, Paolo Perfetti and myself. The case of Cerrina (Fig. 20 ▸) is particularly relevant to our narration and will be expanded on later.

PULS did eventually achieve the transfer of the Frascati synchrotron light research to Adone (Balzarotti *et al.*, 1980 ▸; Belli *et al.*, 1980 ▸), and continued after the end of Adone: it is still active and successful today with activities at the DAΦNE accelerator and elsewhere.

However, the move to Adone did not produce all the potential and positive consequences that were possible because of exceptional favorable circumstances: why?

## 7. The ‘missed opportunity’

Specifically, Frascati did not achieve what should have been its objective: making Italy the absolute world leader in synchrotron light. This was not a crazy dream but a concrete possibility, created by strong advantages over the competition, which included, as we have seen, excellent human resources, a world-leading storage ring, the know-how from the pioneering experiences of ‘Sanità Luce’ and ‘Solidi Roma’ and the strong local condensed-matter scientists.

To better appreciate these advantages, let us make a direct comparison with Tantalus. Frascati had a much more numerous and stronger staff, in particular for accelerators. Plus, Adone was a sophisticated storage ring with high energy – more than 12 times that of Tantalus – so its emitted spectrum extended to much shorter X-ray wavelengths. And, again, Frascati had started synchrotron light activities almost a decade before Tantalus, which, in contrast, was a very small, low-energy facility (se Fig. 16 ▸) with chronically shaky finances. It could not even pay its technicians for around-the-clock operation, so it was stopped during the night. Tantalus was not adequately supported by the parents, the University of Wisconsin and its Physics Department, which did not fully appreciate its potential. Indeed, the local synchrotron users were very few. The Tantalus staff were not numerous and primarily relied on Ed Rowe’s talent, with no backup if he developed a problem (as he later did).

However, these handicaps notwithstanding, Tantalus was undeniably a fantastic success (Margaritondo, 2008 ▸; Lynch *et al.*, 2015 ▸). For years, it was an extremely productive facility where many of the synchrotron light techniques and instruments of today were launched – notably those for surface science and atomic physics – and where many future leaders of synchrotron light facilities around the world received their initial training. Overall, its production and impact were much bigger than those of Adone: so what caused this difference?

The answer is evident: Tantalus was, as we have seen, fully dedicated to synchrotron light – whereas Adone was not. Paradoxically and in spite of their declining importance, particle physics experiments continued to get absolute priority in Frascati. For years, the proposals to use Adone as a light source were rejected or delayed. The PULS experimentalists – of excellent professional stature and with outstanding productivity records – were thus forced to work at Tantalus and at other facilities abroad.

**Figure 19:**
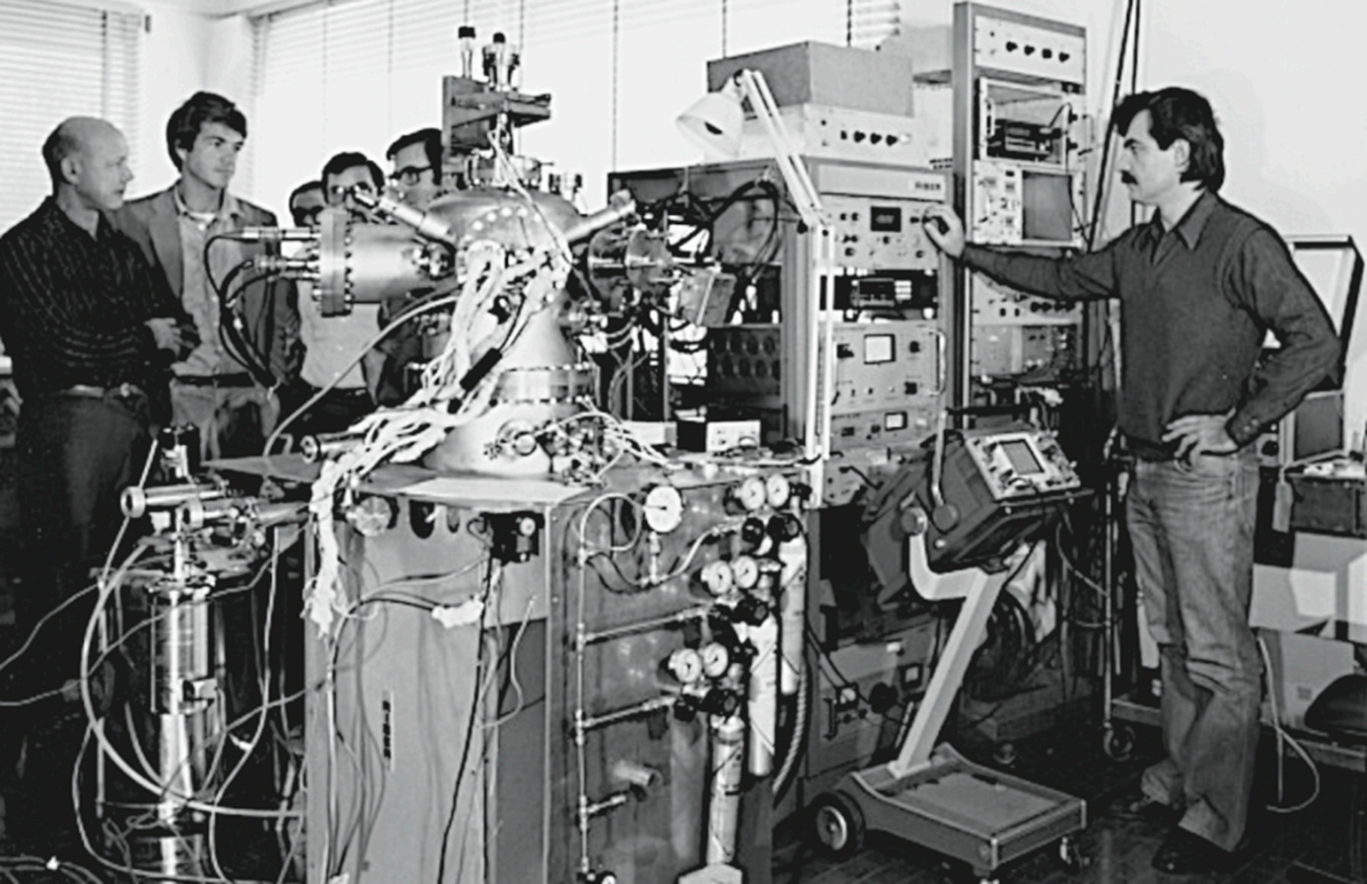
Historic image from the early 1980s: PULS experimental system with several early members of the project. From left to right: Andrzej Kisiel, Stefano Nannarone, Franco Zanini, Francesco Antonangeli, Adolfo Savoia and Franco Cerrina. The figure is an artist’s rendition image created by the author.

**Figure 20:**
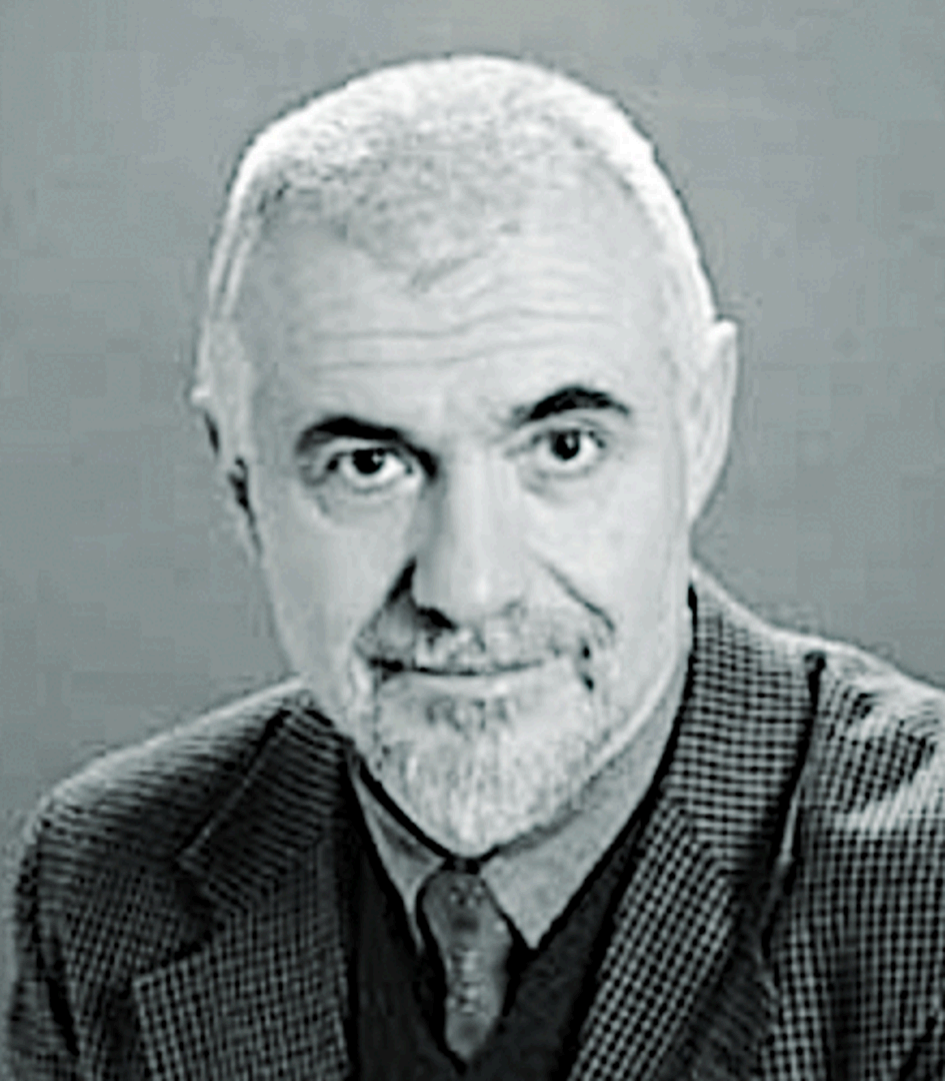
Franco Cerrina, eminent and tragic figure of the Italian synchrotron light diaspora, caused by the ‘missed opportunity’. The image is an artist’s rendition portrait created by the author.

The first synchrotron light experiments with Adone took place (Balzarotti *et al.*, 1980 ▸; Belli *et al.*, 1980 ▸) only in 1979 (Figs. 19 ▸ and 21 ▸), nine years after the inauguration of Tantalus and five after the J/ψ discovery. Furthermore, Adone continued to be shared with particle research and other domains such as nuclear physics, although the PULS scientists confirmed their quality by obtaining from Adone a stream of important results.

The negative consequences of the shared use of Adone are demonstrated by factual data. Consider, for example, those posted by PULS (Preger & Murtas, 1997 ▸) about what it delivered: ‘*120 weeks of beam time dedicated to synchrotron light and 120 weeks used in a parasitic way*’. Here, ‘*parasitic*’ refers to beam time assigned to other activities, which synchrotron light users could exploit only by accepting operation conditions not necessarily optimized for them.

How do the above figures compare with Tantalus? The assessment is complicated since the PULS website does not specify over which time period its beam time was delivered. Let us reasonably assume that it was from the first synchrotron light experiments on Adone in 1979 and until its shutdown in 1993. During the same period, Tantalus – and after 1987 its successor Aladdin – delivered over 700 beam time weeks fully dedicated to synchrotron light. And the Wisconsin center had many more beamlines than Frascati, serving a large number of users.

What should have been done by Frascati to fully exploit its advantages and achieve world leadership? First, after 1974 the INFN should have *immediately* opened Adone to synchrotron radiation experiments – which could have started in 1975 and not in 1979. And then it should have quickly transformed Adone into a dedicated synchrotron source; in particular, by equipping it with several advanced insertion devices (Margaritondo, 2017 ▸) – whereas only one, PWA (Project Wiggler Adone), was realized. Regrettably, the INFN leaders of that time did not see the wisdom of such decisions.

The advantages of Adone and Frascati lasted for about a quinquennium, until the early 1980s. Afterwards, they evaporated since other countries in Europe, North America and Asia developed their own synchrotron facilities – fully dedicated, with strong financial support and with high electron energies, optimized characteristics and advanced equipment. In particular, BESSY I in Berlin and CHESS at Cornell started in 1980, SRS at Daresbury in 1981, NSLS–Brookhaven and the KEK Photon Factory (Tsukuba) in 1982. Italy responded only a decade later, finally realizing in 1993 a dedicated synchrotron source, Elettra in Trieste – thanks to the leadership of the Nobel laureate Carlo Rubbia.

To better understand the five-year opportunity window of Adone, we must consider how synchrotron radiation was evolving worldwide. After the success of Tantalus, several storage rings started to be used as light sources – in particular, ACO in 1973, and in 1974 DORIS in Hamburg, SURF II at the National Bureau of Standards, INS-SOR in Tokyo and SPEAR at SLAC, Stanford.

However, Adone was technically ahead of these competitors, notably because its energy was much higher, except for SPEAR (Doniach *et al.*, 1997 ▸), which could reach 3.7 GeV and could have been the only potential competitor of Adone during its window of opportunity. However, synchrotron light activities at SPEAR were severely handicapped by its parasitic operation, dominated by particle physicists, who, in addition to the usual coexistence problems, imposed an upper energy limit of 1.5–2 GeV – thus impeding synchrotron light experiments with hard X-rays (Doniach *et al.*, 1997 ▸). Therefore, if operated as a fully dedicated light source, Adone with its 3 GeV would have been also ahead of SPEAR.

Why did Frascati not understand and exploit its advantage? The answer is not simple and the historical realities are not entirely clear. However, I can report here a personal experience revealing a surprising fact: there were in Frascati ideas about a full conversion of Adone to synchrotron light as early as 1974.

Indeed, Emilio Burattini elaborated at that time a confidential strategic plan that would have modified the mission of the Laboratori Nazionali. I personally know about this development because Emilio asked my help and we worked together at the project, which envisioned a prominent role for synchrotron light, likely implying a dedicated Adone.

Burattini‘s project remained confidential and, regrettably, led to nothing. Too bad: it could have secured for Italy world leadership in a blossoming key domain of science and technology!

## 8. A heavy price

The consequences of the ‘missed opportunity’ were very severe. Many of the talented synchrotron light experimentalists grown in Frascati emigrated, since the worldwide expansion of the field was creating a very attractive international job market. Some eventually came back to Italy but many did not: the waste of human resources was a national disaster, comparable with the destruction of Fermi’s team.

One special example of this diaspora was Franco Cerrina (Figs. 19 ▸ and 20 ▸), whose story is both extraordinary and tragic. Coming from an indigent family, while studying physics he supported his chronically ill mother working as a technician at the University of Rome I, where he achieved an early, outstanding and now totally forgotten result: the commissioning of *the first laser* in Italy. Purchased from a French company, the device was a piece of junk that did not function at all – until Cerrina fixed its problems with his exceptional technical skills.

After the challenging time as a student-worker, he gained his degree in physics and started a career as a scientist at LESS before the aforementioned transfer to PULS. But later, frustrated by the ‘missed opportunity’, he left Italy to join the team of Jerry Lapeyre (Montana State University), a leader in the use of Tantalus. Cerrina’s professional stature grew rapidly, and he was appointed at a young age to an endowed professorship at the University of Wisconsin-Madison.

His achievements were exceptional. He was the creator and world leading expert of X-ray lithography, a novel technology to fabricate integrated circuits with synchrotron radiation. And he became a top consultant for the American government and for private companies within the ‘Sematech’ consortium. In parallel, he developed *SHADOW*, a worldwide standard software for designing synchrotron beamlines.

Tragically, Cerrina’s life and career ended prematurely in 2010, after he had left Wisconsin for a prestigious chair and directorship in Boston. One morning, he was found dead in his laboratory, apparently due to nitro­gen exposure.

Franco Cerrina is a symbol of all the synchrotron light talents lost by Italy. But his memory was never adequately honored in his country of birth. And I can personally testify that he was discriminated and handicapped in Rome during the first steps of his professional career because of his progressive political ideas – with no consideration for his scientific talent and potential.

## 9. Final comments: why bother?

This is a reasonable question since the events described in this text happened decades ago. And, in a broad prospective, what mattered was not the leadership of Frascati but the worldwide development of synchrotron radiation. So, why deal with obsolete facts with no hope to change their consequences?

The answer is that they are not obsolete at all! Indeed, the effects on Italian science are still felt today, and their causes have not disappeared.

To understand these facts, we must expand a bit our analysis of the ‘missed opportunity’. Why did INFN not make the correct decisions about Adone? After all, its leaders were top-level scientists. And they did downplay weak elementary particle activities, favoring top-quality research.

One important factor in the choices about Adone was the mission of INFN, centered on particle research and not explicitly including service to synchrotron radiation users from other domains. Similar problems, incidentally, influenced the synchrotron radiation role of DOE and NSF in the USA.

A second relevant factor in Italy was a cultural barrier between condensed matter and particle physics. The latter relies on big experiments that often produce individual landmark results. Instead, condensed-matter research is mostly based on systematic studies that explore complex systems with large collections of data, rarely yielding breakthroughs. This second approach is difficult to digest for particle physicists, whose strategies are influenced by the ‘reductionist’ vision of the universe. But it is essential for exploring complicated coordinated systems – and has led to outstanding applications like transistors, integrated circuits, digital computers, LEDs, solar cells and superconducting devices.

The cultural barrier did influence in the 1970s the decisions about Adone (and also SPEAR), which invariantly prioritized particle research targeting big and visible results. And there was a third important factor: the differences in management maturity between elementary particle research and other branches of Italian physics.

Amaldi and his partners had created INFN as an effective alternative, based on merit and highly professional management – essential prerequisites for outstanding successes in particle physics. Unfortunately, the Italian condensed-matter community of the 1970s had not yet reached the same level of management culture. Its centralized organizations like the CNR ‘Gruppo Nazionale di Struttura della Materia’ (GNSM) tried to emulate INFN – but were weaker compromises.

The Italian particle physicists did not feel at ease with this situation: this contributed to the choice not to transfer the full control of Adone to condensed-matter users. Paradoxically, this was one of the few strategic mistakes ever made by the INFN.

We cannot change the historical realities and must live with their consequences, including my personal regret for not having witnessed the world leadership of my country of birth in a key new research domain. Let us hope that the next generations will learn from the ‘missed opportunity’ and avoid similar mistakes. The remarkable but unhappy history reported here could still have a positive impact!

## Figures and Tables

**Figure 11:**
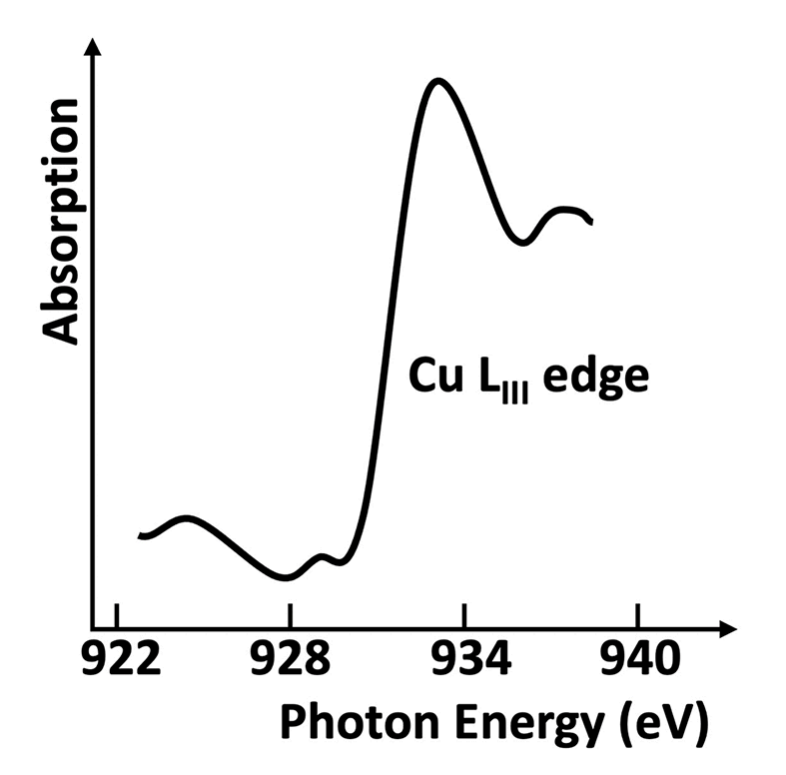
The first results obtained in Frascati by ‘Sanità Luce’ revealed absorption edges. Plot drawn based on data from Cauchois *et al.* (1963*a* ▸,*b* ▸).

**Figure 21:**
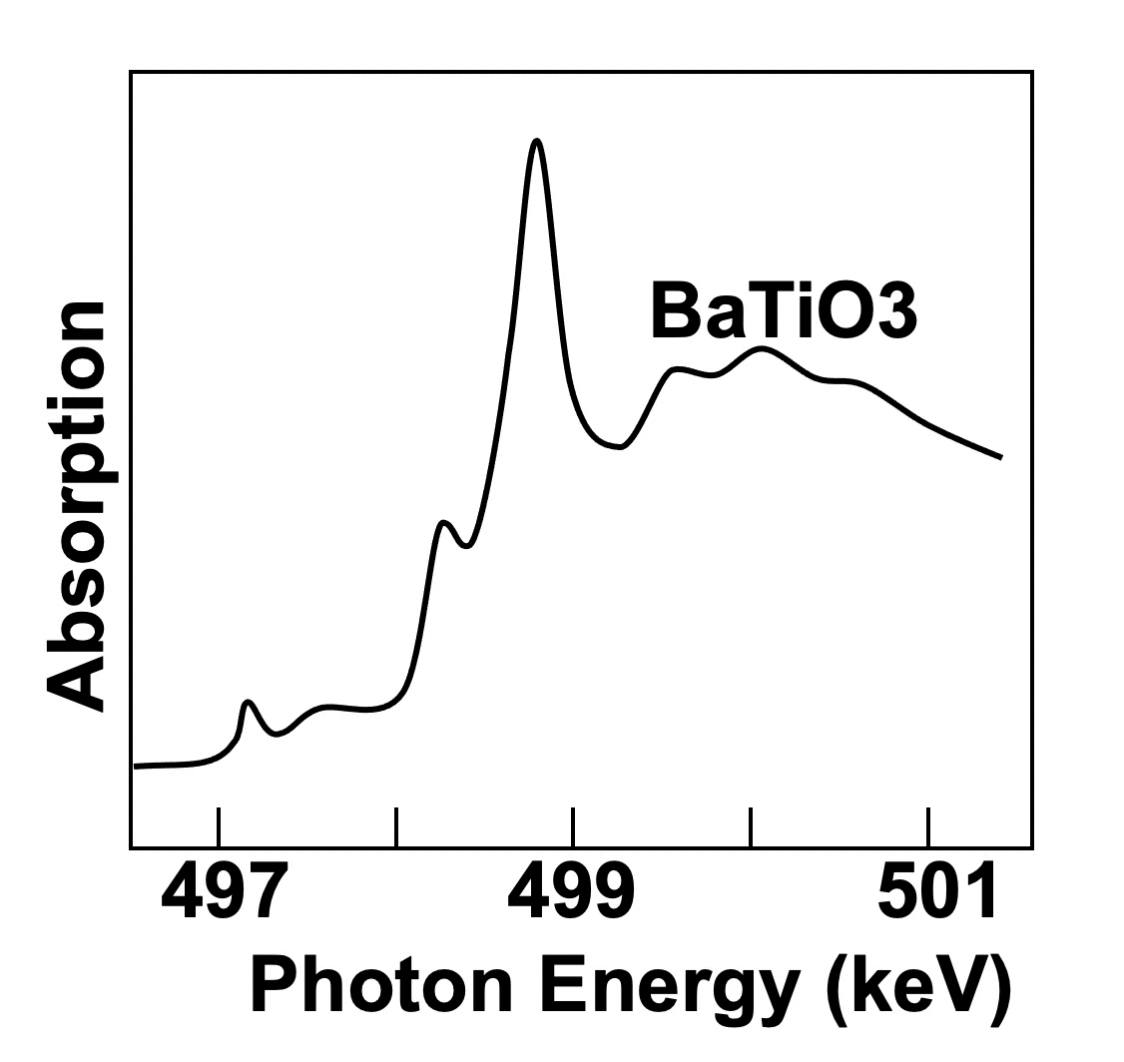
One of the first synchrotron radiation experiments using Adone. The plot was drawn based on data from Balzarotti *et al.* (1980 ▸).

## References

[bb1] Amaldi, E. (1981). *The Bruno Touschek Legacy*, CERN 81-19. CERN, Geneva, Switzerland.

[bb2] Bacci, C., Celio, R. B., Berna-Rodini, M., Caton, G., Del Fabbro, R., Grilli, M., Iarocci, E., Locci, M., Mencuccini, C., Murtas, G. P., Penso, G., Spinetti, G. S. M., Spano, M., Stella, B., Valente, V., Bartoli, B., Bisello, D., Esposito, B., Felicetti, F., Monacelli, P., Nigro, M., Paolufi, L., Peruzzi, I., Mortemi, G. P., Piccolo, M., Ronga, F., Sebastiani, F., Trasatti, L., Vanoli, F., Barbarino, G., Barbiellini, G., Bemporad, C., Biancastelli, R., Cevenini, F., Celvetti, M., Costantini, F., Lariccia, P., Parascandalo, P., Sassi, E., Spencer, C., Tortora, L., Troya, U. & Vitale, S. (1974). *Phys. Rev. Lett.* **33**, 1408–1410.

[bb333] Balzarotti, A., Bartolini, L., Bianconi, A., Burattini, E., Grandolfo, M., Habel, R. & Piacentini, M. (1972). Servizio documentazione LNF 72-98. Laboratori Nazionali di Frascati, Frascati, RM, Italy.

[bb3] Balzarotti, A., Bianconi, A. & Burattini, E. (1974*a*). *Phys. Rev. B*, **9**, 5003–5007.

[bb4] Balzarotti, A., Bianconi, A., Burattini, E., Grandolfo, M., Habel, R. & Piacentini, M. (1974*b*). *Phys. Status Solidi B*, **63**, 77–87.

[bb5] Balzarotti, A., Bianconi, A., Burattini, E. & Strinati, G. (1974*c*). *Solid State Commun.* **15**, 1431–1434.

[bb6] Balzarotti, A., Comin, F., Incoccia, L., Piacentini, M., Mobilio, S. & Savoia, A. (1980). *Solid State Commun.* **35**, 145–149.

[bb7] Balzarotti, A., Piacentini, M. & Grandolfo, M. (1970). *Lett. Nuovo Cimento*, **3**, 15.

[bb8] Belli, A., Scafati, A., Bianconi, A., Mobilio, S., Palladino, L., Reale, A. & Burattini, E. (1980). *Solid State Commun.* **35**, 355–361.

[bb9] Bernardini, C. (2004). *Phys. Persp.* **6**, 156–183.

[bb10] Bernardini, C., Bizzarri, U., Corazza, G. F., Ghigo, G., Querzoli, R. & Touschek, B. (1962). *Nuovo Cimento*, **23**, 202–207.

[bb11] Bernardini, C., Corazza, G. F., Ghigo, G. & Touschek, B. (1960). *Nuovo Cimento*, **18**, 1293–1295.

[bb12] Bernardini, C., Pancheri, G. & Pellegrini, C. (2015). *Rev. Accl. Sci. Tech.* **08**, 269–290.

[bb13] Bonnelle, C. & Dhez, P. (2014). *Hist. Rech. Contemp.* **III**(1), 15–22.

[bb14] Bonolis, L. (2005). *Riv. Nuovo Cim.* **28**(11), 1–60.

[bb15] Bonolis, L., Bossi, F. & Pancheri, G. (2019). *Nuovo Saggiatore*, **37**, 3.

[bb16] Bonolis, L. & Pancheri, G. (2011). *EPJ H*, **36**, 1–61.

[bb17] Burattini, E. (2016). *Synchrotron Radiat. News*, **29**(2), 33–37.

[bb18] Burattini, E., Cossu, E., Di Maggio, C., Gambaccini, M., Indovina, P. L., Marziani, M., Pocek, M., Simeoni, S. & Simonetti, G. (1995). *Radiology*, **195**, 239–244. 10.1148/radiology.195.1.7892478 7892478

[bb19] Burattini, E., Gambaccini, M., Marziani, M. & Rimondi, O. (1992). *Rev. Sci. Instrum.* **63**, 639.

[bb20] Burattini, E. & Simonetti, G. (1992). *Synchrotron Radiat. News*, **5**(5), 12–14.

[bb21] Cauchois, Y., Bonnelle, C. & Missoni, G. (1963*a*). *C. R. Acad. Sci. Paris*, **257**, 409.

[bb121] Cauchois, Y., Bonnelle, C. & Missoni, G. (1963*b*). *C. R. Acad. Sci. Paris*, **257**, 1242.

[bb22] Cimino, R., Burattini, E. & Mobilio, S. (1992). *Rev. Sci. Instrum.* **63**, 1613–1614.

[bb23] Doniach, S., Hodgson, K., Lindau, I., Pianetta, P. & Winick, H. (1997). *J. Synchrotron Rad.* **4**, 380–395. 10.1107/S0909049597012235 16699252

[bb24] Hwu, Y. & Margaritondo, G. (2021). *J. Synchrotron Rad.* **28**, 1014–1029. 10.1107/S1600577521003325 PMC8127362 33950010

[bb25] Iwanenko, D. & Pomeranchuk, I. (1944). *Phys. Rev.* **65**, 343.

[bb26] Jaeglé, P., Carillon, A., Dhez, P., Jamelot, G., Sureau, A. & Cukier, M. (1971). *Phys. Lett. A*, **36**, 167–168.

[bb27] Jaeglé, P., Combet Farnoux, F., Dhez, P., Cremonese, M. & Onori, G. (1968). *Phys. Lett. A*, **26**, 364–365.

[bb28] Jaeglé, P., Missoni, G. & Dhez, P. (1967). *Phys. Rev. Lett.* **18**, 887–888.

[bb29] Kerst, D. W. (1941). *Phys. Rev.* **60**, 47–53.

[bb30] Kisiel, A. (2008). *Synchrotron Radiat. Nat. Sci.* **7**, 10.

[bb31] Lawrence, E. O. & Edlefsen, N. (1930). *Science*, **72**, 376.

[bb32] Lynch, D. W., Plummer, W., Himpsel, F., Chiang, T. C., Margaritondo, G. & Lapeyre, G. J. (2015). *Synchrotron Radiat. News*, **28**(4), 20.

[bb33] Margaritondo, G. (2008). *Phys. Today*, **61**, 37–43.

[bb34] Margaritondo, G. (2017). *Riv. Nuovo Cim.* **40**, 411.

[bb35] Margaritondo, G. (2021). *Quad. Stor. Della Fis.* **25**, 99.

[bb36] Margaritondo, G. (2022). *J. Vac. Sci. Technol. A*, **40**, 033204.

[bb37] Maxwell, J. C. (1865). *Philos. Trans. R. Soc. London*, **155**, 459–512.

[bb38] Maxwell, J. C. (1873). *A Treatise of Electricity and Magnetism.* London: Clarendon.

[bb39] McMillan, E. M. (1945). *Phys. Rev.* **68**, 143–144.

[bb40] Missoni, G. (2017). *Il Laboratorio di Fisica dell’Istituto Superiore di Sanità*, https://www.iss.it/documents/20126/0/Quaderno_12.pdf/5b73b8e9-6203-3782-2d43-0dab5a56fa19?t=1593710943681.

[bb41] Parratt, L. G. (1959). *Rev. Sci. Instrum.* **30**, 297–299.

[bb42] Preger, M. & Murtas, F. (1997). *PULS laboratory*, https://www.lnf.infn.it/esperimenti/puls/adone.html.

[bb43] Savoia, A. (1988). *Synchrotron Radiat. News*, **1**(3), 10–13.

[bb44] Schwinger, J. (1949). *Phys. Rev.* **75**, 1912–1925.

[bb45] Tomboulian, D. H. & Bedo, D. E. (1958). *J. Appl. Phys.* **29**, 804–809.

[bb46] Tomboulian, D. H. & Hartman, P. L. (1956). *Phys. Rev.* **102**, 1423–1447.

[bb47] Tullio, V. & Balerna, A. (2008). *PULS group*, http://www.lnf.infn.it/esperimenti/puls/.

[bb48] Veksler, V. I. (1944). *Proc. USSR Acad. Sci.* **43**, 329.

[bb49] Widerøe, R. (1928). *Arch. Elektrotech.* **21**, 387.

